# Supplementary material for: The protocol for a pilot feasibility trial of Improving Neurodevelopmental ouTcomes After prenatal Cannabinoid in uTero exposure (INTACT) study for a multi-center trial
Source: PLoS One. 2025 May 12;20(5):e0322035. doi: 10.1371/journal.pone.0322035 (PMC12068654; doi:10.1371/journal.pone.0322035)
Supplement: S2 File — The INTACT study protocol. (DOCX) [file pone.0322035.s002.docx]

**Pilot Feasibility Trial of Improving Neurodevelopmental ouTcomes After prenatal Cannabinoid *in uTero* exposure (INTACT)**

**UAMS IRB Protocol Number: 276534**

**National Clinical Trial (NCT) Identified Number: TBD**

**Version Number: V-05**

**Date: 20 September 2024**

**Sponsor: ISPCTN DCOC**

**Funded by: NIH**

**Summary of Key Changes from Version-04 to Version-05:**

| **Affected Section(s)** | **Summary of Revisions Made from V-04 to V-05** | **Rationale** |
| --- | --- | --- |
| Face page and 10.1.6 Key Roles and Study Governance | Replaced XX with XX. | Change in DCOC/overall PI for the study because XX, is leaving UAMS at the end of August 2024. |
| 1.1, Synopsis, Participant Duration | Changed “12-14 months” to “12 months” in the following sentence: “From participant consent of the birthing parent/infant dyad until the child is 12 months of age.” | Given that a month is defined as 28-days for study activities, the infant cannot be 14 months old at the end of the study. |
| 1.3, Schedule of Activities, Participant Activities Table | Added footnote * to month 12 (+/- 7 days of infant age). Removed footnote ^ from month 12 (up to 14 months of infant age). | Clarification |
| 6.1.1, Study Intervention Description | Removed introductory module | There are 11 ePALS modules, not 12 as previously thought. |
| 7.3, Lost to Follow-Up | Added, “Participants will be asked if they are willing to provide names and contact information of back-up contact(s) or other ways for the research team to stay in contact throughout the year-long study. Participants will be informed that once the participant provides back-up/other contact information, the research team may use those method(s) to contact the participant, and that back-up contacts may then learn that the participant is part of this study.” | IRB request for future modification |
| 8.2, 8.3.4, 8.3.5 | Clarified that all AEs related to the intervention will be tracked. | Correction |
| 8.3.6, Serious Adverse Event Reporting | Changed 24 hours to 48 hours in the following sentence: “Reports to the DCOC will be made immediately, within 48 hours of finding out about the event.” | Correction |
| 10.1.6 Key Roles and Study Governance | Added XX and XX as DCOC Co-PIs | Staff change |
| Section 10.1.12, Publication and Data Sharing Policy | Changed “XX” to “XX” in the following sentence: “Other researchers may request data from this trial by contacting XX, at the DCOC.” | Staff change |

**Summary of Key Changes from Version-03 to Version-04:**

| **Affected Section(s)** | **Summary of Revisions Made from V-03 to V-04** | **Rationale** |
| --- | --- | --- |
| 2.3.3, Assessment of Potential Risks and benefits | Replaced “The trial has minimal possible risks, and the potential benefit of improved birthing parent responsiveness, which can then improve infant outcomes. The study is covered by a certificate of confidentiality automatically granted to all NIH-funded research that collects or uses identifiable and/or sensitive information. Therefore, the trial's potential benefits outweigh the potential risks” with “The trial has greater than minimal risk due to possible legal/social consequences for participants if confidentiality were to be breached. The study is covered by a certificate of confidentiality automatically granted to all NIH-funded research that collects or uses identifiable and/or sensitive information. With additional precautions to be implemented locally (section 2.3.1), we feel that the trial's potential benefit of improved birthing parent responsiveness, which can then improve infant outcomes, outweighs the potential risks.” | cIRB determination of greater than minimal risk |
| 8.2, Safety and Other Assessments | Replaced “given the minimal risk of the study” with “given the minimal biological/medical risk of the study” | cIRB determination of greater than minimal risk |
| 10.1.1.2, Consent Procedures and Documentation | Deleted “While this study is minimal risk,” from the beginning of the sentence that now reads, “The study team will collect informed written consent and Health Insurance Portability and Accountability Act (HIPAA) authorization.” | cIRB determination of greater than minimal risk |

**Summary of Key Changes from Version-02 to Version-03:**

| **Affected Section(s)** | **Summary of Revisions Made from V-02 to V-03** | **Rationale** |
| --- | --- | --- |
| 1.1, 3, 8.1, 9.1, 9.2 | Removed “completed the first coaching session” from objective-1/endpoint-1 description. | The population for Objective 1 was reverted to what was originally submitted to the Data Safety Monitoring Board. “Completed the first coaching session” was removed to prevent artificially influencing the outcomes. |
| 1.1, 3, 8.1, 9.1, 9.2 | Clarified that the coaching session when the child is 12 months old is the final session, and that at least 14 participants are needed to meet the 70% threshold for Objective 2. | Clarification was needed. |
| 1.1, 3, 8.1, 9.1, 9.2 | Clarified that the number of coaching sessions completed will be counted to determine if the session threshold has been met, and that at least 16 participants are needed to meet the 80% threshold of participants who meet the session threshold for Objective 3. | Clarification was needed. |
| 9.3, Populations for Analysis | Changed “consented dyads with confirmed eligibility” to “that which is approached for study participation” in the following sentence: “The population for analysis for objective 1 is that which is approached for study participation.” Added, “The population for analysis for objectives 2 and 3 is consented dyads with confirmed eligibility.” | Clarification was needed. |
| 1.2 Schema | The specification of “obstetric” clinics was removed. | Sites may recruit from other types of clinics as well. |
| 1.3, 4.1 | Specified activities that may be completed up to 7 days after infant birth/delivery: obtaining informed consent and Cannabinoid use Survey. | This was added to specify a maximum window after birth when a participant may be recruited for participation. |
| 1.3, 4.1, | Defined one month as a 28 day period for scheduled study activities. | Changed to standardize participant activity windows. |
| 2.2, 6.1.1 | Changed “PALS/ePALS” and “PALS” to “ePALS” only where this term relates to the INTACT intervention. | cIRB review contingency |
| 2.3.1 Known Potential Risks | Added the following text:  “Each participating site will seek the local protections available in its state. This may include a state-issued Memorandum of Understanding, Memorandum of Agreement, institutional waivers for medical record documentation requirements for research studies, and/or other mechanisms. Each participating site will discuss available protections with local legal counsel to minimize risk to participants. Potential participants will be notified of these protections and associated risks in the local-context consent form. The DCOC will obtain documentation that local legal counsel has reviewed local context information added to the consent form template. Documentation may be in the form of a letter or email from local legal counsel or may be included in local IRB acknowledgement of local context language.” | cIRB review contingency |
| 4.1 Overall Design | Added underlined text to the following sentence: “The duration of the study for each birthing parent/infant dyad is 12 months, with all interactions with study staff after the delivery hospitalization being completed remotely.” | Interactions with study staff may be in person during the delivery hospitalization but will be completed remotely after the delivery hospitalization. |
| 4.1 Overall Design | Changed “the delivery hospitalization” to “after infant birth” at the end of the following sentence: “Screening may begin in the 3^rd^ trimester; however, consent and the Cannabinoid Use Survey will not be completed until after infant birth.” | Consent and the Cannabinoid Use Survey will be completed after infant birth and mostly during the delivery hospitalization, not after the delivery hospitalization. |
| 4.1 Overall Design | Removed the sentence, “All demographic information will be collected during the delivery hospitalization.” | It is possible that demographic information will be collected after the delivery hospitalization. |
| 5.1 Inclusion Criteria | Changed “We anticipate that 20 birthing parent/infant dyads will be consented, assigned to the intervention, and complete at least the first coaching session” to “We anticipate that 20 birthing parent/infant dyads will be approached, consented, and determined to be eligible for study participation.” | Aligns with changes to endpoint descriptions. |
| 5.5 Strategies for Recruitment and Retention, Inpatient Recruitment | Removed, “Potential participants will be approached by the study team members to introduce the study, gauge interest, and confirm eligibility” and replaced it with, “Potential participants referred by the clinical care team will be approached by study team members to introduce the study and confirm eligibility. Study team members are embedded within the clinical care team and will help providers identify potential participants prior to engagement.” | cIRB review contingency |
| 5.5 Strategies for Recruitment and Retention, Participant Retention | Changed “the birth hospitalization study tasks“ to “study orientation” in the following sentence: “Additionally, an incentive will be provided upon completion of study orientation.” | Ties compensation to one activity that may or may not occur in the hospital. |
| 6.1.1 Study Intervention Description | Added the sentence, “The content of the modules is the same as previously used and published by the ePALS group with different populations of interest. The timing of the modules (1 per month) and the population of interest are what is changed for this study.” | cIRB review contingency |
| 6.1.1 Study Intervention Description | Added the sentence, “As there will be interaction with the infant during the sessions, the infants are expected to be present during the coaching session.” | cIRB review contingency |
| 6.1.1 Study Intervention Description | Reduced the minimum length of time between sessions from 3 weeks to 2 weeks. | Due to the ±7-day window and the 28-day month standardization, there might not be 3 whole weeks between sessions. |
| 7.1 Discontinuation of Study Intervention | Removed the sentence, “If either dyad participant has a prolonged hospitalization of ≥ 3 months during the study period, the study intervention will be discontinued.” | The study team decided we do not need to specify this discontinuation criteria. |
| 8.2 Safety and Other Assessments | Clarified that, “All reported AEs will be evaluated for severity, and the study team will track all severe and serious AEs,” instead of only those that are potentially study related. | We will only be tracking and reporting severe AEs and SAEs for this study. |
| 8.3.4 Time Period and Frequency for Event Assessment and Follow-up | Removed the sentence, “Participants may report severe AEs for seven days after completing all study procedures and SAEs for 30 days after completing all study procedures.” | Changed due to the very limited medical risk to participants. |
| 9.3 Statistical Analysis | Added the sentence, “At baseline, demographics will be collected.” | cIRB review contingency |
| 9.3 Statistical Analysis | Removed the text, “Numerical variables will also be inspected for outliers and empirical distributions during data review. Methods for handling outliers and skewed data will be defined and described in the SAP. For all variables collected in this study, including survey scales and their item scales, we will use the simple linear regression model or Pearson’s correlation coefficient to assess the relationship between numerical variables; the one-way ANOVA model to assess the association of numerical dependent variables to a categorical independent variable; and the Chi-square test to assess the association of two categorical variables. Item reliability of a survey instrument will be assessed of Cronbach's alpha if it hasn’t been used in populations similar to this study. Such statistical plans can also help monitor the quality of data collection and provide preliminary knowledge of the importance of clinical variables to a future study when a full clinical trial is conducted.” | cIRB review contingency |
| 9.4.4 Safety Analysis | Clarified that, “We will detail all severe AEs and SAEs experienced by the birthing parent/infant dyad,” instead of only those that are treatment related. | We will be tracking and reporting all severe AEs and SAEs for this study. |
| 10.1.3 Confidentiality and Privacy | Added underlined text to the following sentence: “Data will be de-identified before sharing externally; data sent from the individual sites to the DCOC will be coded information and have no identifiers.” | cIRB review contingency |
| 10.1.10 Data Collection and Management Responsibilities | Added the sentence, “Data will be de-identified before sharing externally; data sent from the individual sites to the DCOC will be coded information and have no identifiers.” | cIRB review contingency |

**Summary of Key Changes from Version-01 to Version-02:**

| **Affected Section(s)** | **Summary of Revisions Made from V-01 to V-02** | **Rationale** |
| --- | --- | --- |
| 1.1, Synopsis; 3, Objectives and Endpoints; 9.1, Statistical Hypothesis | Objective 2 hypothesis – deleted “of the randomized participants.” | There is no randomization. |
| 2.1, 2.2, 2.3, 3, 4.1, 4.2, 5.1, 5.2, 5.4, 5.5, 6.1, 8.1, 9.4.4, 10.1.1.2 | Replaced the term “caregiver” with “birthing parent” throughout protocol, only where it applies to the study and not where it refers to literature. | cIRB pre-review contingency |
| 2.3.1, Known Potential Risks | Added, “There is a risk that someone outside the study could learn that the participant used marijuana during pregnancy. To mitigate this risk,”  Added that the risk mitigation includes a Certificate of Confidentiality, “Additionally, this research is covered by a Certificate of Confidentiality (CoC) from the National Institutes of Health. This Certificate of Confidentiality is automatically granted to all NIH-funded research and protects the privacy of research participants by prohibiting disclosure of identifiable, sensitive information from research records to anyone not connected to the research or as specified in the informed consent document.” | cIRB pre-review contingency |
| 4.1, Overall Design | Edited the 3^rd^ paragraph to describe the rationale for the final screening (Cannabinoid Use Survey) being completed after obtaining the participant’s consent to join the study. Added the following:   - “Research coordinators will contact potential participants to briefly describe the study and perform a preliminary eligibility screen using the Initial Contact Script.”   “Participants may feel more comfortable providing this information after the study has been described in detail, with the added assurance of confidentiality from the CoC and the HIPAA authorization. Responses to the survey will be retained in the electronic data capture (EDC) system, linked to the participant ID code.” | cIRB pre-review contingency |
| 5.1, Inclusion Criteria | Inclusion criteria for birthing parents, 4^th^ bullet – “Delivers” changed to “Birth parent who delivers.”  Inclusion criteria for infants, 2^nd^ bullet added – “Biological child of the birthing parent” | cIRB pre-review contingency |
| 5.5, Strategies for Recruitment and Retention | Added to the Outpatient Recruitment section that, “Possible strategies for the use of social media will not involve potential participants uploading anything on social media sites that would indicate they may have used marijuana during pregnancy.” | cIRB pre-review contingency |
| 5.5, Strategies for Recruitment and Retention | The Consort Diagram was edited to show a branching point for participants that withdraw consent prior to initiation of the study intervention. An allocation step was added and the “First session of intervention completed” step was removed. The objective information boxes were also removed. | The updated consort diagram more accurately describes the population of interest for all three objectives. |
| 6.1.1, Study Intervention Description | Removed the study-wide backup coach (7^th^ coach). | The study team no longer plans on using a 7^th^ coach. |
| 7.1, Discontinuation of Study Intervention | Clarified that, “If a birthing parent participant loses their device that they use to access study activities for a period of ≥3 consecutive coaching sessions, the study intervention will be discontinued.” | cIRB pre-review contingency |
| 7.2, Participant Discontinuation/Withdrawal from the Study | Clarified that, “Site can also replace the birthing parent/infant dyad if they withdraw from the study before beginning the first month’s coaching session.” | These participants will not be included in our analysis and can be replaced on the study. |
| 7.3, Lost to Follow-Up | Clarified that only private messing will be used on social media for re-contact purposes. | cIRB pre-review contingency |
| 8.1, Efficacy Assessments | Objective 1 – clarified that the target number of participants who complete the first coaching session will be compared to the number of participants approached. | The population of interest for the first objective is those approached, given the objective is participant recruitment. |
| 8.3.4, Time Period and Frequency for Event Assessment and Follow-up; 8.3.5, Adverse Event Reporting | Clarified that all severe AEs and SAEs will be collected and reported, whether or not they are related to study procedures. | To ensure participant safety and minimize confusion for our research staff, we will report all severe AEs and SAEs. |
| 9.2, Sample Size Determination | Objective 1 – deleted “consenting approaching up to 100 birthing parent/infant dyads whose eligibility will be confirmed by the Cannabinoid Use Survey. Among them.”  Clarified that our recruitment goal is 20% of the approached population. | The number of participants approached cannot be known at this time. |
| 9.3, Populations for Analyses | The defined population for analysis was redefined as “consented dyads with confirmed eligibility.” | This definition more accurately describes the population for all three objectives. |
| 10.1.1, Informed Consent Process | Added entire paragraph, “To meet study objectives, we will consent up to 50 participant dyads. Birthing parent participants will then complete the Cannabinoid Use Survey for the final eligibility screen. Up to 20 eligible participant dyads will be assigned to the intervention, not including participants that withdraw from the study before beginning the first month’s coaching session. Recruitment will continue until one of these targets has been met (consent or intervention assignment).” | The statistical considerations (Section 9) for all study outcomes do not rely on numbers of consented/enrolled participants, so these regulatory expectations have been added to this section. |

**Table of Contents**

[Statement of Compliance 13](#_Toc161824336)

[1 Protocol Summary 14](#_Toc161824337)

[1.1 Synopsis 14](#_Toc161824338)

[1.2 Schema 16](#_Toc161824339)

[1.3 Schedule of Activities (SoA) 17](#_Toc161824340)

[2 Introduction 18](#_Toc161824341)

[2.1 Study Rationale 18](#_Toc161824342)

[2.2 Background 19](#_Toc161824343)

[2.3 Risk/Benefit Assessment 23](#_Toc161824344)

[2.3.1 Known Potential Risks 23](#_Toc161824345)

[2.3.2 Known Potential Benefits 24](#_Toc161824346)

[2.3.3 Assessment of Potential Risks and Benefits 24](#_Toc161824347)

[3 Objectives and Endpoints 25](#_Toc161824348)

[4 Study Design 26](#_Toc161824349)

[4.1 Overall Design 26](#_Toc161824350)

[4.2 Scientific Rationale for Study Design 27](#_Toc161824351)

[4.3 End of Study Definition 28](#_Toc161824352)

[5 Study Population 29](#_Toc161824353)

[5.1 Inclusion Criteria 29](#_Toc161824354)

[5.2 Exclusion Criteria 29](#_Toc161824355)

[5.3 Lifestyle Considerations 30](#_Toc161824356)

[5.4 Screen Failures 30](#_Toc161824357)

[5.5 Strategies for Recruitment and Retention 30](#_Toc161824358)

[6 Study Intervention 34](#_Toc161824359)

[6.1 Study Intervention(s) Administration 34](#_Toc161824360)

[6.1.1 Study Intervention Description 34](#_Toc161824361)

[6.2 Preparation/Handling/Storage/Accountability 35](#_Toc161824362)

[6.2.1 Acquisition and accountability 35](#_Toc161824363)

[6.2.2 Appearance, Packaging, and Labeling 35](#_Toc161824364)

[6.3 Measures to Minimize Bias: Randomization and Blinding 35](#_Toc161824365)

[6.4 Study Intervention Compliance 35](#_Toc161824366)

[7 Study Intervention Discontinuation and Participant Discontinuation/Withdrawal 36](#_Toc161824367)

[7.1 Discontinuation of Study Intervention 36](#_Toc161824368)

[7.2 Participant Discontinuation/Withdrawal from the Study 36](#_Toc161824369)

[7.3 Lost to Follow-Up 36](#_Toc161824370)

[8 Study Assessments and Procedures 37](#_Toc161824371)

[8.1 Efficacy Assessments 37](#_Toc161824372)

[8.2 Safety and Other Assessments 38](#_Toc161824373)

[8.3 Adverse Events and Serious Adverse Events 38](#_Toc161824374)

[8.3.1 Definition of Adverse Events (AE) 38](#_Toc161824375)

[8.3.2 Definition of Serious Adverse Events (SAE) 38](#_Toc161824376)

[8.3.3 Classification of an Adverse Event 38](#_Toc161824377)

[8.3.4 Time Period and Frequency for Event Assessment and Follow-Up 39](#_Toc161824378)

[8.3.5 Adverse Event Reporting 40](#_Toc161824379)

[8.3.6 Serious Adverse Event Reporting 40](#_Toc161824380)

[8.3.7 Reporting Events to Participants 41](#_Toc161824381)

[8.3.8 Events of Special Interest 41](#_Toc161824382)

[8.3.9 Reporting of Pregnancy 41](#_Toc161824383)

[8.4 Unanticipated Problems 41](#_Toc161824384)

[8.4.1 Definition of Unanticipated Problems (UP) 41](#_Toc161824385)

[8.4.2 Unanticipated Problem Reporting 42](#_Toc161824386)

[8.4.3 Reporting Unanticipated Problems to Participants 42](#_Toc161824387)

[9 Statistical Considerations 43](#_Toc161824388)

[9.1 Statistical Hypotheses 43](#_Toc161824389)

[9.2 Sample Size Determination 44](#_Toc161824390)

[9.3 Populations for Analyses 44](#_Toc161824391)

[9.4 Statistical Analyses 44](#_Toc161824392)

[9.4.1 General Approach 44](#_Toc161824393)

[9.4.2 Analysis of the Primary Efficacy Endpoint(s) 45](#_Toc161824394)

[9.4.4 Safety Analyses 45](#_Toc161824395)

[9.4.5 Baseline Descriptive Statistics 45](#_Toc161824396)

[9.4.8 Tabulation of Individual Participant Data 45](#_Toc161824397)

[10 Supporting Documentation and Operational Considerations 46](#_Toc161824398)

[10.1 Regulatory, Ethical, and Study Oversight Considerations 46](#_Toc161824399)

[10.1.1 Informed Consent Process 46](#_Toc161824400)

[10.1.2 Study Discontinuation and Closure 47](#_Toc161824401)

[10.1.3 Confidentiality and Privacy 48](#_Toc161824402)

[10.1.4 Multi-site Communications (IRB-related) 49](#_Toc161824403)

[10.1.5 Future Use of Stored Specimens and Data 49](#_Toc161824404)

[10.1.6 Key Roles and Study Governance 50](#_Toc161824405)

[10.1.7 Safety Oversight 50](#_Toc161824406)

[10.1.8 Clinical Monitoring 51](#_Toc161824407)

[10.1.9 Quality Assurance and Quality Control 51](#_Toc161824408)

[10.1.10 Data Handling and Record Keeping 51](#_Toc161824409)

[10.1.11 Protocol Deviations 52](#_Toc161824410)

[10.1.12 Publication and Data Sharing Policy 53](#_Toc161824411)

[10.1.13 Conflict of Interest Policy 53](#_Toc161824412)

[10.2 Additional Considerations 53](#_Toc161824413)

[10.3 Abbreviations 53](#_Toc161824414)

[11 References 56](#_Toc161824415)

# Statement of Compliance

The trial will be carried out in accordance with International Conference on Harmonization Good Clinical Practice (ICH GCP) and the following:

- United States (US) Code of Federal Regulations (CFR) applicable to clinical studies (45 CFR Part 46, 21 CFR Part 50, 21 CFR Part 56, 21 CFR Part 312, and/or 21 CFR Part 812)

National Institutes of Health (NIH)-funded investigators and clinical trial site staff who are responsible for the conduct, management, or oversight of NIH-funded clinical trials have completed Human Subjects Protection and ICH GCP Training.

The protocol, informed consent form(s), recruitment materials, and all participant materials will be submitted to the Institutional Review Board (IRB) for review and approval.  Approval of both the protocol and the consent form must be obtained before any participant is enrolled.  Any amendment to the protocol will require review and approval by the IRB before the changes are implemented to the study.  In addition, all changes to the consent form will be IRB-approved; a determination will be made regarding whether a new consent needs to be obtained from participants who provided consent, using a previously approved consent form.

# 1 Protocol Summary

## 1.1 Synopsis

| **Title:** | Pilot Feasibility Trial of Improving Neurodevelopmental ouTcomes After prenatal Cannabinoid *in* *uTero* exposure (INTACT) |
| --- | --- |
| **Study Description:** | This is a multisite pilot study to test the feasibility of studying a novel program, the INTACT Intervention, to train birthing parents in contingent responding with the goal of improving neurodevelopmental outcomes in infants prenatally exposed to cannabinoids. We will examine the effectiveness of 1) recruitment rates, 2) completion rates, and 3) the INTACT intervention number of coaching sessions completed. Birthing parents are defined as the biological parent who gave birth to the infant. |
| **Objectives:** | \| **Primary Objective:** Conduct a rigorous feasibility pilot study of a novel program to improve neurodevelopmental outcomes in infants prenatally exposed to cannabinoids as the basis for a future randomized controlled trial. We will conduct the feasibility trial in three ISPCTN sites to assess the following variables: participant recruitment, participant completion when the infant is 12 months of age, and completion of overall INTACT intervention coaching sessions. This information will inform a future randomized controlled trial, at which time a clinical outcome assessment with the Bayley Scales of Infant and Toddler Development Screening Test, 4^th^ Edition (Bayley-IV) will be completed at 2 years of age. Thus, no clinical outcome assessment will be completed in this feasibility pilot study.  **Objective 1: Participant Recruitment:** To examine if birthing parents can be recruited and enrolled in the study with multi-faceted recruitment approaches, which may include distributing flyers in the obstetrical and family medicine clinics (for individuals with self-report) and utilizing electronic health record (EHR) review to identify and approach pregnant and post-partum individuals with positive toxicology screen(s). Providers may give flyers individually to birthing parents in the obstetrical and family medicine clinics if the individual uses cannabinoids in pregnancy. **Hypothesis:** The percentage of participants eligible for study participation will be greater than 20% of those approached, consistent with other similar feasibility studies.  **Objective 2: Participant Completion:** To evaluate participant completion, as measured by the percent of birthing parent/infant dyads that complete the coaching session scheduled when the child is 12 months of age (last coaching session; recruitment goal of n=20 dyads). **Hypothesis**: This study will have participant completion of at least 70% (n≥14 dyads) at the final visit when the child is 12 months of age.  **Objective 3: Participant Adherence:** To evaluate the number of individual INTACT intervention coaching sessions completed. This will be measured by evaluating the number of sessions completed. **Hypothesis**: 80% of the participants (n≥16) will receive a sufficient number of INTACT intervention coaching sessions, defined as at least 8 of the 12 sessions.^1^ \| \| --- \| |
| **Endpoints:** | \| Primary Endpoints:   1. Whether a potential participant approached for study participation will eventually be consented and determined to be eligible for study participation. 2. Whether a birthing parent/infant dyad will complete the final coaching session when the child is 12 months of age. 3. Whether a participant will complete the sufficient number of INTACT intervention coaching sessions, defined as completion of at least 8 out of 12 coaching sessions. \|  \| \| --- \| --- \| |
| **Study Population:** | Sample size n=20 (6-7 participants per site, 3 Institutional Development Award (IDeA) States Pediatric Clinical Trials Network [ISPCTN] sites). |
| **Phase:** | Pilot |
| **Description of Sites/Facilities Enrolling Participants:** | The study team will conduct the INTACT trial at three ISPCTN site-awardee states. The study team will enroll participants into the INTACT trial from one hospital per ISPCTN-awardee state. |
| **Description of Study Intervention:** | The study intervention is described below:   - The birthing parent/infant dyad will complete 12 monthly INTACT intervention coaching sessions, each of which consists of online module completion followed by a personalized coaching session. Individuals will complete the online module each month prior to the coaching session with an interventionist. - Each of the online modules contains information to strengthen effective parenting practices that promote early language, cognitive, and social development. Parenting skills that are introduced in the online module will be reinforced with the monthly coaching session. - During the monthly coaching sessions, the coaches will guide the birthing parent on contingent responding, which includes “reading infant signals, responding with warm and sensitive behaviors, maintaining infants’ focus of attention, watching for opportunities to introduce an object or social game, using rich verbal content in combination with physical demonstrations and incorporating this constellation of behaviors in everyday activities”.^1^ |
| **Study Duration:** | The study will last for 22 months: 1-4 months for local IRB approval as needed, 1-4 months for interventionist training as well as clinical coordinator training at each study site, 3 months for enrollment, with follow-up of 12 months per participant dyad, and 3 months for data organization, clean-up, and manuscript preparation, as well as submission of a proposal for the conduct of a full-scale clinical trial based on study results.    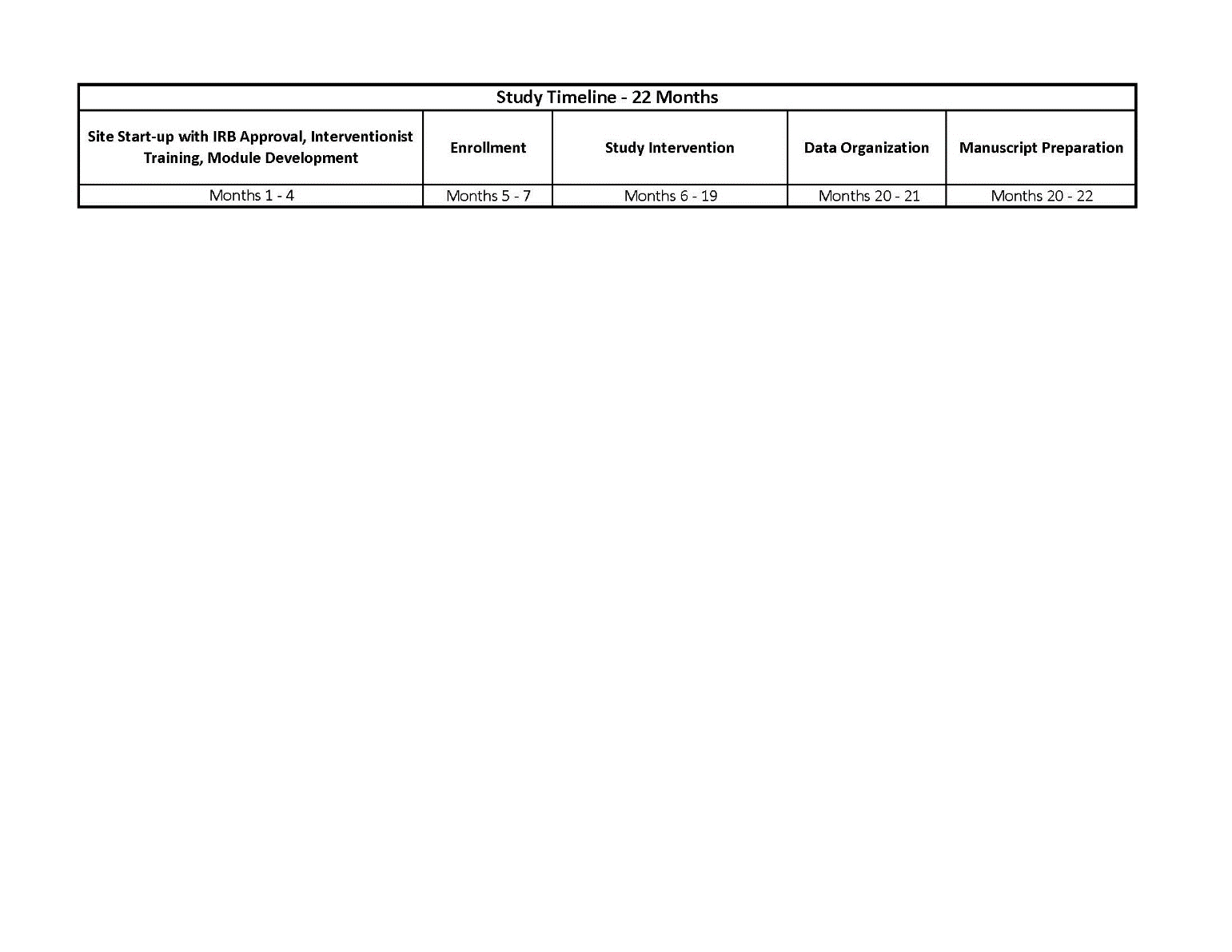 |
| **Participant Duration:** | From participant consent of the birthing parent/infant dyad until the child is 12 months of age. |

## 1.2 Schema

| 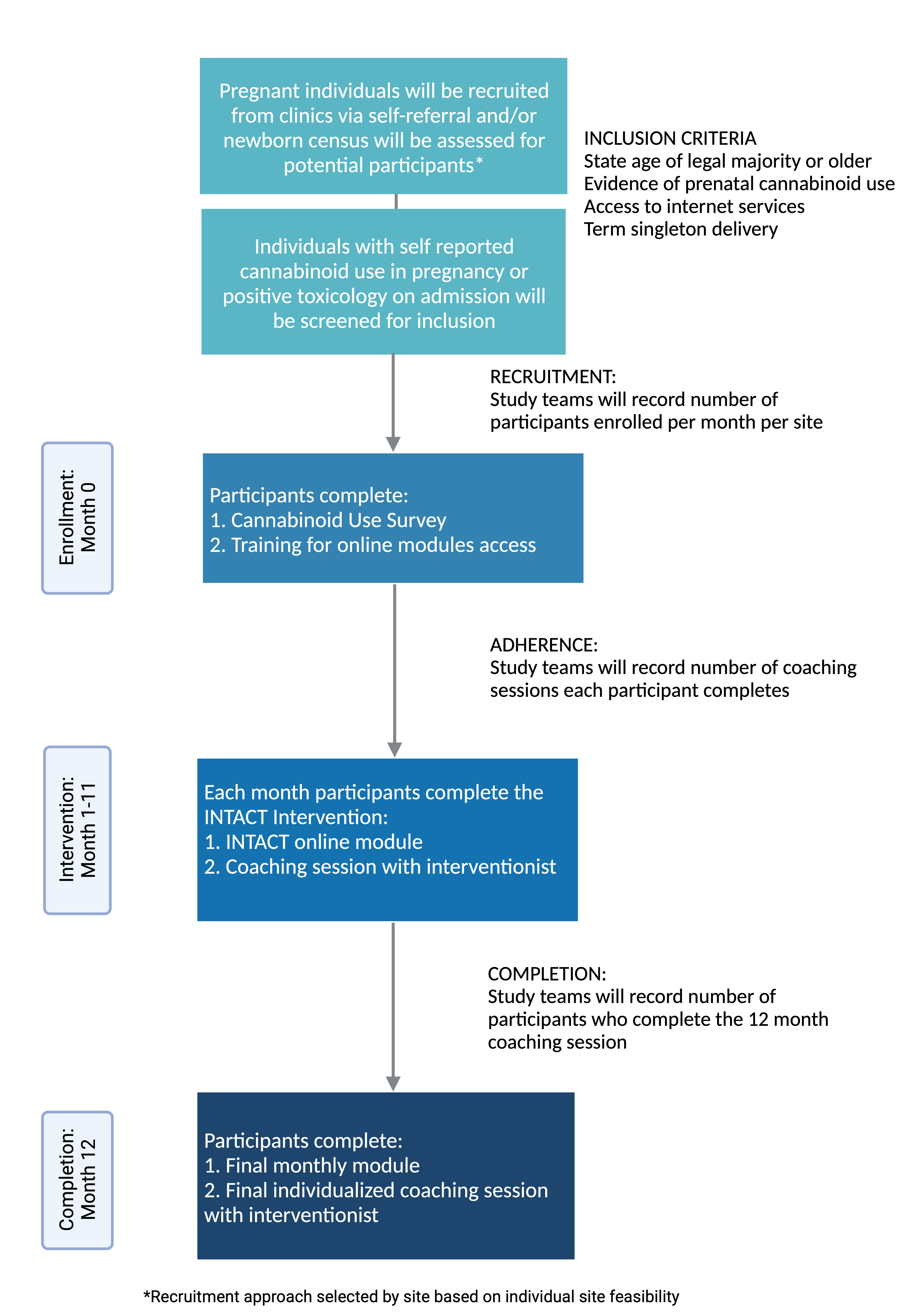 |
| --- |

## 1.3 Schedule of Activities (SoA)


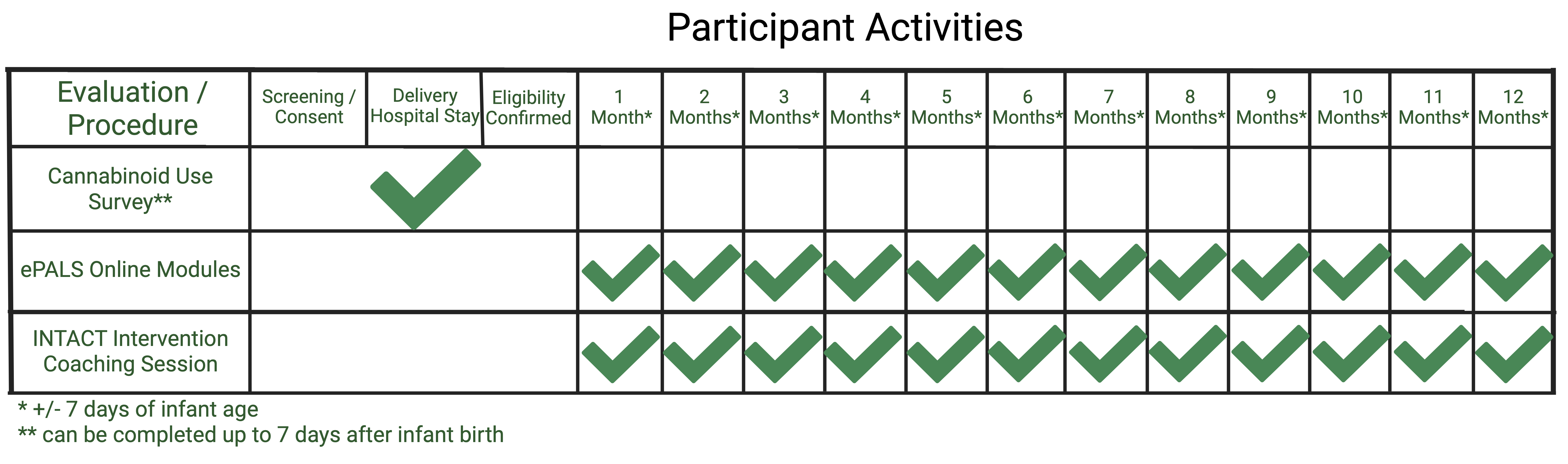
The above table shows the activities that the participants will complete in relation to the infant’s age. Consent will be obtained prior to completion of the cannabinoid use survey. Each month is defined as a 28-day period.

# 2 Introduction

## 2.1 Study Rationale

Studies have shown a risk of neurodevelopmental issues in children born to individuals using cannabinoids during pregnancy.^2-10^ As more states continue to legalize the recreational use of cannabinoids, it becomes prudent to provide early intervention to infants exposed *in utero* to cannabinoids so that these babies have the best opportunity for normal neurodevelopment. Contingent parenting, which will be taught through the INTACT intervention, has the potential to promote normal developmental trajectories for high-risk children, such as those with intrauterine cannabinoid exposure. Contingent parenting includes “reading infant signals, responding with warm and sensitive behaviors, maintaining infants’ focus of attention, watching for opportunities to introduce an object or social game, using rich verbal content in combination with physical demonstrations and incorporating this constellation of behaviors in everyday activities”.^1,11^ Early intervention services are federally funded grant programs for children with disabilities from birth through 21 years old. While these services can be accessed by infants exposed to cannabinoids prenatally who subsequently demonstrate neurodevelopmental delay, early intervention services are secondary prevention, only accessible to children once they are identified as delayed or with clear risk factors for delay. Unfortunately, prenatal cannabinoid exposure is not a qualifying risk factor for early intervention services. Additionally, early intervention services focus on teaching skills to the infant/child, with no guidance provided to the caretaker. By contrast, the INTACT intervention will be offered for all infant participants, regardless of whether delays are noted at the initiation of the intervention. In addition, the intended focus will be on birthing parent behaviors and contingent caregiving, which in turn strengthens the trust and bond between the child and birthing parent. The intended purpose is to maximize the opportunity for the child to experience a safe and secure home environment in which to explore and develop to facilitate higher levels of learning. As this network remains committed to positive child health, pre-, peri-, and post-natal health, and neurodevelopmental outcomes, this study addresses multiple focus areas within the ISPCTN. Additionally, there is no competing network to accomplish this study.

We propose to conduct a pilot feasibility trial at three sites within the ISPCTN to examine 1) recruitment rates, 2) completion rates when the infant is 12 months of age, and 3) individual INTACT intervention coaching sessions completion rates. In the development of this protocol, we have included investigators from across the country that reside in states with different legal statuses of cannabinoid use to ensure all aspects of cannabinoid legality that could affect study enrollment and participation were considered. The writing protocol team includes Dr. Jessie Maxwell from New Mexico (recreational cannabinoid use was just legalized in 2021 and effective April 2022), Dr. Leigh-Anne Cioffredi from Vermont (recreational cannabinoid use has been legal since 2018), and Dr. Maria Barber from South Dakota (recreational cannabinoid use remains illegal). The goal of this writing team is to include participants living in states that represent the spectrum of legalization status that is currently observed across the country (please see Figure 1 in the Background Section for a map of the legalization status at all ISPCTN awardee states).

As this pilot study examines the feasibility of implementing this innovative intervention as well as the feasibility of recruitment and study completion, it will provide formative data that will be used to design a future fully powered randomized controlled trial (RCT) to test the efficacy of the INTACT intervention. Key elements this study will address that are integral to inform a larger RCT include: 1) feasibility of recruiting from this vulnerable, high-risk population and estimations of recruitment rate; 2) ability for participants to complete the coaching session when the infant is 12 months of age; and 3) ability to engage participants sufficiently in the intervention with appropriate adherence. With the growing number of states across the country continuing to legalize recreational use of cannabinoids, it is important to identify interventions that have the potential to ensure strong neurodevelopmental outcomes among infants exposed to cannabinoids prenatally. The ISPCTN has experience in conducting clinical trials in high-risk populations, such as opioid-exposed infants in the two ongoing ACT NOW trials. We will utilize this expertise to ensure similar success in this pilot trial. Additionally, the interventions are not typically available in the more remote areas of the country, so completion of this trial in the ISPCTN allows for a high-risk population to have access to additional interventions.

## 2.2 Background


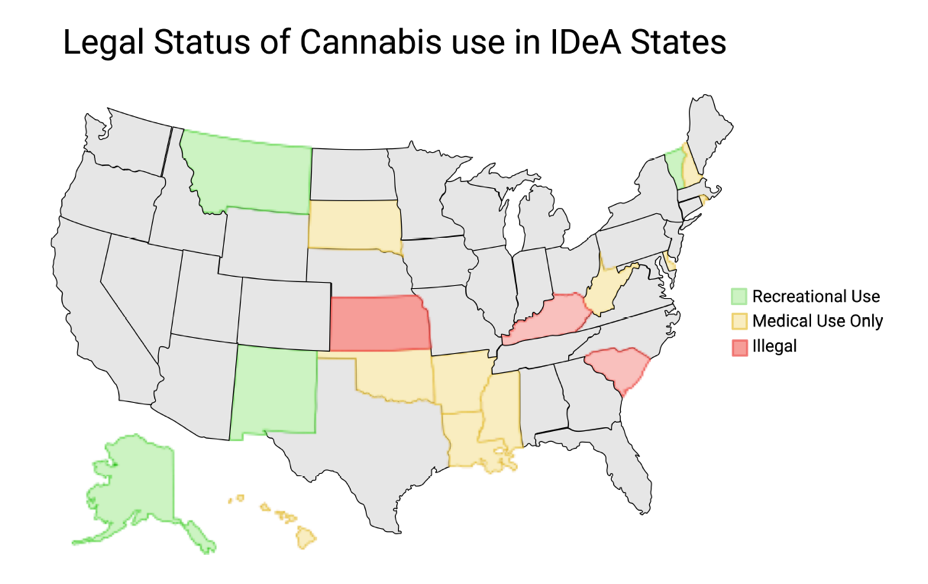
Cannabinoids represent the third most frequently used substance during pregnancy, behind alcohol and tobacco.^12^ Additionally, the recent movement to legalize the recreational use of cannabinoids has been coupled with increasing use during pregnancy. At the time of submission of this proposal, 18 states and the District of Columbia have legalized recreational cannabinoid use and the Marijuana Opportunity Reinvestment and Expungement (MORE) Act, ending the federal ban on cannabinoids, was passed by the House of Representatives on April 1, 2022. Figure 1 shows the legalization status of ISPCTN awardee states. Within this social context, significant changes in cannabinoid use have been reported. According to the 2019 National Household Survey on Drug Use and Health, 5.4% of pregnant individuals in the United States used cannabinoids in the past month. This rate is 28% higher than use just ten years earlier.^13^ Not only is the prevalence of prenatal cannabinoid use increasing, recent studies indicate rates of daily use and the quantity used during pregnancy is higher than previously established by cohort studies which enrolled in the early 1980s.^14^

**Figure 1:** Legalization status of cannabinoid in ISPCTN sites.

More widespread and frequent use of cannabinoids during pregnancy is occurring as concentrations of Δ^9^‑tetrahydrocannabinol (THC), the psychoactive ingredient in marijuana, is also increasing in marijuana and its products. Eloshly et al. reported a 300% increase in the concentration of THC in recreational cannabinoid products since the 1990s.^15,16^ This increase in potency, quantity, and prevalence of cannabinoid use in pregnancy suggests there is a substantial increase in fetal exposure to THC.

The potential for neurodevelopmental impacts due to intrauterine THC exposure is high. THC is a highly lipophilic molecule that readily crosses the placenta and has been shown to accumulate in fetal and placental tissue.^17,18^ Moreover, the endogenous endocannabinoid system (ECS) is known to play a critical role in early brain development, in particular effecting proteins involved in axonal growth and neuronal connectivity.^19-21^ Evidence shows the primary brain cannabinoid receptor (CB_1_) is most intensely expressed in the human fetal brain in the mesocorticolimbic system, with particular predominance in the amygdala and hippocampus. In fact, expression of CB_1_ is highest during gestation and drops precipitously after birth, indicating mid-gestation may be the developmental period of highest susceptibility to exposure to THC.^22^ This growing body of evidence is complemented by emerging evidence that fetal neurotransmitter activity and neuronal connectivity are altered in those with prenatal cannabinoid exposure. Specifically, Wang et al. reported altered dopamine and opioid receptors in mid-gestation fetuses with prenatal cannabinoid exposure.^23,24^ Additionally, Thomason et al. described fetal functional magnetic resonance imaging (fMRI) findings that suggested altered hippocampal connectivity in fetuses with prenatal cannabinoid exposure.^25^

The theoretical and growing body of evidence describing fetal brain changes associated with prenatal cannabinoid exposure indicates that updated studies are needed to describe the effects of current levels of THC and resultant intrauterine exposure on infant developmental outcomes. The upcoming Heathy Brain Child Development Study (HBCD), enrolling pregnant women in the next 3 years, will be well poised to address the need for investigation of the impact of current cannabinoid use on infants. Extant literature evaluating the impact of prenatal cannabinoid exposure on development during infancy includes three prospective longitudinal studies: the Ottawa Prenatal Prospective Study (OPPS), the Maternal Health Practices and Child Development Study (MHPCD), and the Generation R study.^26-28^ Importantly, two of these enrolled participants in the late 1970s and early 1980s when exposures were vastly different than today. These studies are complemented by three other longitudinal cohorts, the Adolescent Brain Cognitive Development Study (ABCD), Lifestyle and Early Achievement in Families Study (LEAF), and the Norwegian Mothers and Child Cohort Study (MoBa), which investigate the effects of prenatal cannabinoid exposure on neurodevelopment in childhood, but do not report developmental outcomes in the first 1-2 years of life. A recent systemic review and meta-analysis that included these longitudinal studies found overwhelming evidence of detrimental neonatal outcomes from prenatal cannabinoid exposure that included: (1) low birth weight <2500g, (2) preterm birth, (3) Newborn Intensive Care Unit (NICU) admission, (4) small for gestational age (SGA) status, (5) low Apgar score at 1 minute of life and (6) small head circumference; all of which reflect abnormal fetal development.^29,30^ Based on SGA status alone, a recent meta-analysis found poorer cognitive function during the first 12 years of life in children born SGA compared to appropriate for gestational age (AGA) matched for gestational age children.^31^

In addition to differences in birth outcomes, the OPPS study found that intrauterine exposure to cannabinoids was highly associated with an increase in exaggerated startle reflex and tremors with a significantly diminished responsiveness to light in the neonatal period.^26,32^ Altered sleep patterns were found in the MHPCD study with a trend toward increased irritability in those infants with prenatal cannabinoid exposure.^27^ The MHPCD cohort also demonstrated higher amounts of cannabinoid use (>1 joint/day) during the 3^rd^ trimester, which was associated with decreased mental scores of the Bayley Scales of Infant Development Scale at 9 months of age, a difference that disappeared at 18 months age.^33^ In contrast, the Generation R study found evidence of increased aggression and inattention in 18-month-old girls.^34^ These longitudinal studies also found executive functions altered by intrauterine exposure to cannabinoids, including attention, planning, vocalization, and working memory.^6,35^ All together, these cognitive and behavioral functions are critically important for problem-solving situations that require integration and manipulation of basic visuoperceptual skills (see Figure 2).^36^ Thus, there is significant
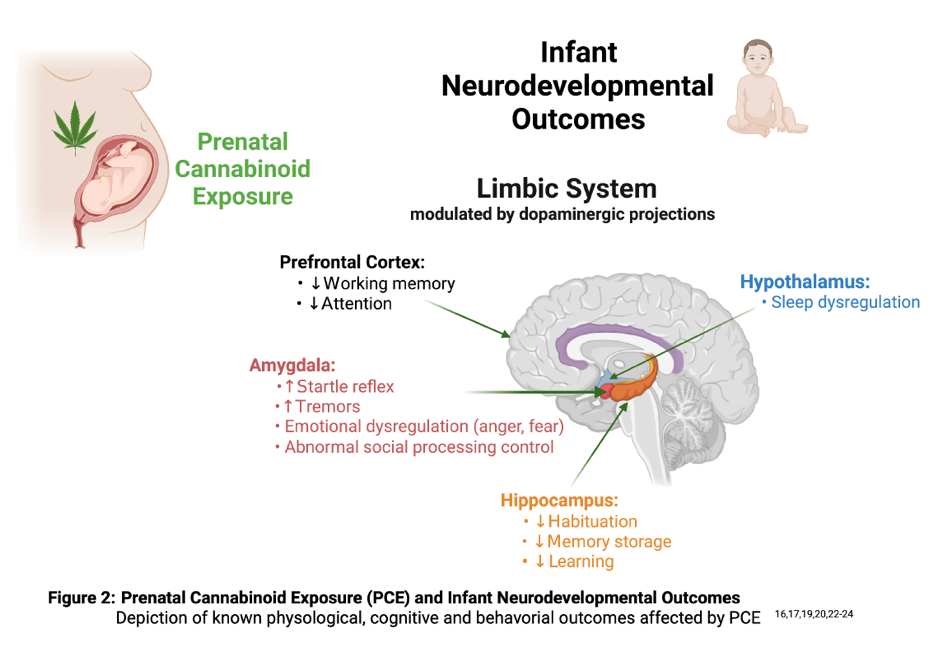
evidence to suggest that prenatal cannabinoid exposure has deleterious effects on the developing brain and significant potential to directly impact infant neurodevelopment.

However, intrauterine exposure to cannabinoids is only one exposure within a larger number of risk factors associated with altered infant neurodevelopment. Throughout gestation, maternal environmental factors (such as maternal depression, smoking, and nutrition) influence the cellular maturation and differentiation as well as the epigenome of the fetus resulting in exposure-influenced phenotypes.^37^ After delivery, the early childhood environment is associated with differences in infant development.^38^ A wealth of research on child development has established the strongly beneficial effects of adaptive and healthy parenting styles on normative development throughout all stages of childhood, with infancy being one of the most influential periods.^38^ One of the strongest and most consistent predictors of infant neurodevelopmental outcomes is the quality of interactions they have with their caregivers. Caregiver responsiveness, defined as timely, sensitive reactions to infant cues,^39^ has been repeatedly associated with positive language, social emotional, and cognitive development.^40-43^

In addition to the neurobiological hit that infants experience following intrauterine exposure to cannabinoids, they are also more likely to be exposed to harsh parenting and less sensitive parenting.^44,45^ In fact, some hypothesize that it is precisely the presence of post-natal stressors that act as the “second hit” following prenatal cannabinoid exposure that precipitates meaningful changes in development.^46^ Results supporting this hypothesis have been reported by Eiden and colleagues,^38,44,47^ who conducted a prospective observational study involving 97 mother-child dyads with prenatal cannabinoid and tobacco co-exposure and 69 unexposed dyads. In both groups, mother-child interactions were observed during infancy and evaluated for levels of maternal harshness and sensitivity through observation of warmth and positive affect. In addition, mothers completed measures of anger and depression, and children completed tasks at age 2 years measuring autonomic and emotion regulation. Finally, maternal reports of child behavior problems were assessed at age 2. Results showed that mothers of cannabinoid-tobacco-exposed infants reported significantly higher levels of anger and depression, and exhibited significantly higher levels of harshness and lower levels of sensitivity during mother-infant interactions compared to mothers of unexposed infants. In turn, levels of maternal harshness during mother-infant interaction were inversely associated with toddler autonomic regulation, and levels of maternal sensitivity were positively associated with toddler emotion regulation. Finally, maternal sensitivity during a child’s infancy was inversely associated with child behavior problems at age 2 years. The findings of these important studies suggest that mothers who use cannabinoids prenatally may be more likely to exhibit maladaptive interaction patterns with their infants characterized by overly harsh and insensitive responses to infant behavioral cues. Such interaction patterns are predictive of subsequent deficits in toddler self-regulation and behavioral problems.

**INTACT will use the PALS/ePALS intervention.** This presents an opportunity for intervention to enhance the post-natal environment by strengthening the foundational building blocks of infant development, specifically the infant-caregiver relationship. For INTACT, the caregiver is the birthing parent. Caregiver sensitivity and responsiveness to their infants, or contingent responding, has a positive impact on infant outcomes. The evidence-based Play and Learning Strategies (PALS) program, a rigorously tested intervention^48,49^, has been shown to strengthen maternal responsiveness and sensitivity which, in turn, protects against child maltreatment and improves infant social-emotional behavior and developmental outcomes.^41,48-51^ This program has demonstrated significant increases in multiple responsiveness behaviors that facilitate infants’ growth in social, emotional, communication, and cognitive competence. Specifically, PALS consists of intervention sessions in which an interventionist/coach uses the curriculum in a flexible manner to meet the individual caregiver’s learning needs. Each PALS session is up to 1 hour in duration and includes scripted didactic, probing questions, video segment sharing to illustrate each concept, and points for discussion.^48,50^ The PALS curriculum has since been adapted to a virtual platform, the ePALS program.^1^

The PALS/ePALS intervention has previously been utilized among mothers whose infants were born at very low birth weights, mothers with depressive symptoms, mothers from low-income families, mothers whose infants were born extremely premature, and socially disadvantaged mothers.^41,48-51^ Results have included increased word use in the infant, an increase in attention in the infant, and an increase in positive behavior during the caregiver-child interaction by both participants. Evidence suggests the PALS/ePALS program impacts children’s behaviors through a change in caregiver responsiveness behavior and improved infant social and emotional skills. Caregivers who have participated in PALS/ePALS have shown an increase in verbal scaffolding (ability to add tailored support to the infant to aide in learning) among participants, and their children have greater gains in cognitive scores among their infants as compared to caregivers and infants who did not receive the intervention.^41,49-52^ With the recent pandemic, interventions requiring in-home visitation have either not occurred or been significantly reduced. Thus, this protocol proposes using the ePALS intervention, which has expanded the reach of the evidence-based PALS intervention by utilizing technology (video conferencing and online learning management platforms) to promote social-emotional development in early childhood.^1^

In terms of measuring the effect of contingent response training for caregivers (birthing parents in INTACT) on early infant development, there is no gold standard, validated measure of neurodevelopmental outcomes for infants younger than 2 years of age. At 2 years of age, the Bayley Scales of Infant and Toddler Development Screening test, 4^th^ Edition (Bayley-IV), can be used as a validated screening test to determine if a child is meeting typical developmental milestones or if any additional comprehensive assessments are required. Thus, the randomized controlled trial to be completed after the pilot study would include the Bayley-IV test as the clinical outcome. As the ISPCTN is utilizing the Bayley-IV in two current ongoing studies, this component is not needed for this feasibility trial.

**Summary.** Collectively, findings from the research discussed above underscore the myriad of developmental challenges facing infants with prenatal cannabinoid exposure stemming from possible *in-utero* neurologic changes, as well as potential ongoing environmental threats stemming from suboptimal birthing parent-child interactions. Evidence suggests early intervention focusing on improving caregiving skills among birthing parents of infants exposed prenatally to cannabinoids may help to offset or counteract delayed infant neurodevelopment.^53,54^ Caregiver training interventions have been shown to improve caregiver skills and behavior, caregiver-infant interaction quality, and infant developmental outcomes among caregivers with depression from low-income settings.^1,11,55,56^ Thus, the INTACT intervention will be focused on birthing parent interventions modeled on ePALS with the goal of improving the infant developmental outcomes of this highly vulnerable population.

## 2.3 Risk/Benefit Assessment

### 2.3.1 Known Potential Risks

Study risks include discomfort or boredom with completing the online modules and coaching sessions and potential loss of confidentiality related to all data collection. There is a risk that someone outside the study could learn that the participant used marijuana during pregnancy. To mitigate this risk, the protocol study team will institute measures to protect the privacy of medical information, including the coding of all HIPAA identifiers from medical records, limitation of access to the medical records to research personnel, and removal of any individual identifiers in reports and publications generated from the study. Additionally, this research is covered by a Certificate of Confidentiality (CoC) from the National Institutes of Health. This Certificate of Confidentiality is automatically granted to all NIH-funded research and protects the privacy of research participants by prohibiting disclosure of identifiable, sensitive information from research records to anyone not connected to the research or as specified in the informed consent document. Each participating site will seek the local protections available in its state. This may include a state-issued Memorandum of Understanding, Memorandum of Agreement, institutional waivers for medical record documentation requirements for research studies, and/or other mechanisms. Each participating site will discuss available protections with local legal counsel to minimize risk to participants. Potential participants will be notified of these protections and their limitations in the local-context consent form. The DCOC will obtain documentation that local legal counsel has reviewed local context information added to the consent form template. Documentation may be in the form of a letter or email from local legal counsel or may be included in local IRB acknowledgement of local context language.

There will be no additional toxicology screening (birthing parent or infant) performed beyond what medical professionals would typically obtain as part of usual institutional care at the site.

While unlikely, completing the cannabinoid use survey and completion of the modules and coaching sessions could be distressing to participants. At the time of enrollment, participants will be provided a card with informational resources to support their wellbeing. This includes mental health information and social service information, including the following:

- SAMSHA National Help Line: 1-800-662-4357
  - SAMHSA’s National Help Line is a free, confidential, 24/7, 365-day-a-year treatment referral and information service (in English and Spanish) for individuals and families facing mental and/or substance use disorders.
- National Domestic Violence Hotline: 1-800-799-7233
  - Advocates are available 24/7, 365-day-a-year to talk confidentially with anyone experiencing domestic violence, seeking resources or information, or questioning unhealthy aspects of their relationship.
- National Suicide and Crisis Lifeline: 988 OR 1-800-273-8255
  - The National Suicide Prevention Lifeline is a national network of local crisis centers that provides free and confidential emotional support to people in suicidal crisis or emotional distress 24 hours a day, 7 days a week.

If any concerns arise for postpartum depression in the birthing parent during the study period, the birthing parent will again be provided with national hotline support numbers as listed above and encouraged to follow up with their primary care provider. Each site may add local mental health resources as well to the cards that are provided to the birthing parent participant. As the study assessments and intervention should not illicit postpartum depression concerns, the above information will be provided to those individuals who volunteer any information concerning postpartum depression.

For concerns regarding child maltreatment, the study personnel will follow state laws for reporting suspected child abuse/maltreatment.

### 2.3.2 Known Potential Benefits

The infants in the study may or may not benefit directly from participation. The primary potential benefits of trial participation include improved birthing parent responsiveness, which can positively impact infant outcomes and development.

### 2.3.3 Assessment of Potential Risks and Benefits

The trial has greater than minimal risk due to possible legal/social consequences for participants if confidentiality were to be breached. The study is covered by a certificate of confidentiality automatically granted to all NIH-funded research that collects or uses identifiable and/or sensitive information. With additional precautions to be implemented locally (section 2.3.1), we feel that the trial's potential benefit of improved birthing parent responsiveness, which can then improve infant outcomes, outweighs the potential risks.

# 3 Objectives and Endpoints

| PRIMARY  OBJECTIVES | ENDPOINTS | JUSTIFICATION FOR ENDPOINT |
| --- | --- | --- |
| **Objective 1 Participant Recruitment:** To examine if birthing parents can be recruited and will be eligible for the study with multi-faceted recruitment approaches, which may include distributing flyers in the obstetrical and family medicine clinics (for individuals with self-report) and use of EHR review to identify and approach pregnant and post-partum individuals with positive toxicology screen(s). Providers may give flyers to individual birthing parents in obstetrical and family medicine clinics if the individual uses cannabinoids in pregnancy.  **Hypothesis**: The percentage of participants eligible will be greater than 20% of those approached, consistent with other similar feasibility studies. | Whether a potential participant approached for study participation will eventually be eligible for study participation. | The 20% recruitment rate is considered feasible to inform a multi-site (18 sites) study in the future. |
| **Objective 2: Participant Completion:** To evaluate participant completion, as measured by the percent of birthing parent/infant dyads that complete the final coaching session scheduled when the child is 12 months of age (recruitment goal of n=20 dyads).  **Hypothesis**: This study will have participation completion of at least 70% (n≥14) at the final coaching visit when the child is 12 months of age. | Whether a birthing parent/infant dyad will complete the final coaching session when the child is 12 months of age. | Based on estimates from the 2021 Neonatology Research Network (NRN) retention data, in addition to ISPCTN trial experience (iAmHealthy), 60-90% long-term follow-up is feasible with infant populations. As this study includes a high-risk population, we will test the feasibility of longer-term follow-up with at least 70% of the participant dyads completing the month 12 coaching session. |
| **Objective 3. Participant Adherence:** To evaluate patient adherence as the number of individual INTACT intervention coaching sessions completed. This will be measured by the number of sessions completed.  **Hypothesis**: 80% (n≥16) of the participants will receive a sufficient number of INTACT intervention coaching sessions completed, defined as at least 8 of the 12 sessions.^1^ | Whether a participant will complete the sufficient number of INTACT intervention coaching sessions, defined as completion of at least 8 out of 12 coaching sessions. | The number of coaching sessions needed to obtain an effective exposure is based on prior ePALS studies (see Background section).^1^ |

# 4 Study Design

## 4.1 Overall Design

The feasibility of recruitment and study completion with individuals who used cannabinoids at any point during pregnancy in a clinical trial at three sites within the ISPCTN will be evaluated. Furthermore, the feasibility of deploying the INTACT intervention, which consists of training birthing parents in contingent responding through individualized coaching sessions tailored for each birthing parent by individual access to asynchronous online learning, will be evaluated.

The duration of the study for each birthing parent/infant dyad is 12 months, with all interactions with study staff after the delivery hospitalization being completed remotely. Please see the Schedule of Activities in Section 1.3 for the overall study timeline and specific intervention and survey timeline. Each month is defined as a 28-day period.

Birthing parents with a positive urine toxicology screen for THC or self-reported cannabinoid use during pregnancy will be recruited for participation. Individuals can be referred by healthcare providers, self-refer during 3^rd^ trimester obstetrical appointments after viewing flyers placed in the obstetrical and family medicine clinics, or be recruited by study staff during the birth hospitalization. Providers may give flyers individually to birthing parents in the obstetrical and family medicine clinics if the patient uses cannabinoids during the 2^nd^ and 3^rd^ trimesters in pregnancy. Individuals with a positive toxicology report for THC during the birth hospitalization will be identified by an automated daily chart review of the electronic medical records with results sent to the local study team; this automated report will ensure that the study team is aware of admissions as a prescreen for potentially eligible individuals. A partial HIPAA waiver will be obtained for pre-screening procedures used to identify potential participants. Birthing parents with a positive toxicology screen for THC or who self-report use of cannabinoids will be considered for participation in the study. Research coordinators will contact potential participants to briefly describe the study and perform a preliminary eligibility screen using the Initial Contact Script. All birthing parent use of cannabinoids will be confirmed with the Cannabinoid Use Survey only after the individual consents to study participation. Participants may feel more comfortable providing this information after the study has been described in detail, with the added assurance of confidentiality from the CoC and the HIPAA authorization. Responses to the survey will be retained in the electronic data capture (EDC) system, linked to the participant ID code.

Consent will not be obtained until after the birth of the infant to ensure that inclusion / exclusion criteria are met, and thus the cannabinoid use survey will not be completed until after the birth of the infant. For individuals that indicated interest in the study prenatally, their due date will be noted so that study staff can monitor the hospital patient census for their delivery.

The birthing parent/infant dyads (goal n=20) will be recruited either before labor or after delivery, depending on the methods of recruitment. Screening may begin in the 3^rd^ trimester; however, consent and the Cannabinoid Use Survey will not be completed until after infant birth; those who consent to participate and meet eligibility criteria will all receive the INTACT intervention as there is no control group for this feasibility study. Individuals may sign consent once the infant has been born to ensure that the infant meets all inclusion/exclusion criteria. Within seven days of delivery, consent will be obtained followed by completion of the Cannabinoid Use Survey. Those meeting inclusion/exclusion criteria will receive instructions for the online sessions, and ensure they have access to complete a video phone call (having access to items such as a smart phone or laptop, etc).

Participants will be contacted via telephone within 3 weeks of giving birth to schedule the first coaching session via a cloud-based video conferencing service. Each month thereafter, the participant will complete the following two activities: 1) viewing that month’s asynchronous online module, which will provide instruction to the participant on key components of contingent parental responses; and 2) coaching session with a trained interventionist/coach who can provide tailored feedback and advice to the participant while focusing on the specific aspect of contingent parenting covered by that month’s online module. The participants will be provided access to one online module each month (12 total, including the introductory module) that they will be instructed to complete prior to the next coaching session. These INTACT intervention modules (described in the “Intervention” section) will provide instructions to the participants to support contingent caregiving. A total of 12 coaching sessions (averaging 1 per month) will occur for the participants.

The following survey will be used as stated (please see Schedule of Activities in Section 1.3):

- Cannabinoid Use Survey to be completed after consent and within 7 days of delivery

The Cannabinoid Use Survey was adapted from the survey being used by the ECHO cohorts and the Early Life Exposures Assessment Tool (ELEAT).^57^ The best measures to identify pregnant people who use cannabinoids during pregnancy is a debated topic in the current literature. Multiple health organizations, including the American College of Obstetricians and Gynecologists (ACOG) and the World Health Organization (WHO), recommend screening for substance use in pregnancy early during prenatal care visits.^58^ Universal drug screening is not recommended by any of these groups specifically, and verbal screening (non-structured requests for self-report) is favored by ACOG and the American Society of Addiction Medicine (ASAM).^59^ These recommendations are reflected in the clinical standard of care across the US, in which the majority of substance use in pregnancy is identified by self-report. Even so, there is significant evidence that self-report underestimates the prevalence of substance use, including cannabinoid use.^60^ Interestingly, evidence also demonstrates one-time toxicological screening also significantly underestimates cannabinoid use in pregnancy.^61^ Historical cohort studies, including Maternal Health Practices and Child Development (MHPCD), the Ottawa Prenatal Prospective Study (OPPS), and Generation R (Gen R) studies have relied on maternal self-report to identify and quantify cannabis use, all demonstrating differences in neurodevelopmental outcomes of offspring using these methods. Gen R obtained urine specimens *and* self-report measures in a subset of participants and found that 71% of participants with cannabinoid use identified by self-report *or* toxicology and had reported their cannabinoid use to their healthcare provider.^61^ Therefore, since self-report has good specificity for identifying cannabinoid use and theoretically may cause fewer feelings of stigmatization and more participant comfort/autonomy, we will use self-report to assess prenatal cannabinoid exposure in this study.

At 12 months of infant age, the number of coaching sessions completed will be collected with the purpose of accurately describing the intervention implementation.

## 4.2 Scientific Rationale for Study Design

This is an open-labeled study with all participants completing the intervention. This approach is being taken to allow for feasibility testing, including the feasibility of delivering the INTACT Intervention with fidelity, so that a larger scale trial can be designed upon completion of this study. Once feasibility has been established with this study, a larger-scale trial would include a control group. As this is a short (12 months of birthing parent participation) feasibility pilot trial, we intentionally are not collecting clinical outcomes; the future RCT would include a post-intervention assessment of birthing parent/infant interaction quality, and assessment of infant neurodevelopment at 2 years of age via administration of the Bayley Scales of Infant and Toddler Development Screening Test, 4^th^ Edition (Bayley-IV). As the Bayley-IV is currently a tool being utilized successfully by ISPCTN sites for a separate trial’s outcome assessment, testing the feasibility of administering the Bayley-IV test within an ISPCTN study context is not needed. Currently, the network is completing a 2-year follow-up with the high-risk population of infants with prenatal exposure to opiates, and this study duration is 2 years total; thus, we feel that testing the feasibility of study completion at 1 year is sufficient for this pilot study.

## 4.3 End of Study Definition

The trial will end for each birthing parent/infant dyad when they have: 1) completed the 12-month intervention (as shown in the Participant Schedule of Activities in Section 1.3), 2) been declared lost to follow-up, or 3) officially withdrawn from the trial.

# 5 Study Population

## 5.1 Inclusion Criteria

We anticipate that 20 birthing parent/infant dyads will be approached, consented, and determined to be eligible for study participation. As the Cannabinoid Use Survey will be completed after consent is obtained and may result in individuals no longer meeting inclusion / exclusion criteria, we expect the total number of individuals consented will be higher than the number determined to be eligible. Participants will come from three different clinical sites with a goal of 6-7 dyads per site that meet all inclusion / exclusion criteria.

Inclusion criteria for **birthing parents** are as follows:

- Age of majority, as defined by the state of residency
- Cannabinoid use during pregnancy confirmed with self-report
- Have the ability to speak, read, and understand English
- Birthing parent who delivered at one of the hospitals where study team members have clinical privileges to access medical records
- Has parental custody of the infant
- Singleton pregnancy with live birth
- Has an electronic device capable of watching videos and able to stream/download videos for viewing and permitting video conferencing (such as a smart phone, laptop, etc.)
- Has steady access to the internet

Inclusion criteria for **infants** are as follows:

- Term infants at birth (≥37 weeks’ gestation)
- Biological child of the birthing parent

## 5.2 Exclusion Criteria

As the goal of this study is to inform a trial to improve the outcome of infants with prenatal cannabinoid exposure, we are excluding birthing parents with multi-substance use to minimize confounding factors (tobacco and alcohol are not considered multi-substance use for the study). Consent will not occur until after the infant is born to ensure the following are not present:

Exclusion criteria for **birthing parents** are as follows:

- Other illicit drug use, excluding cannabinoids, during pregnancy (such as heroin and cocaine) per self-report or toxicology results
- Opiate use (prescribed or unprescribed) per self-report or toxicology results during this pregnancy
- Prolonged hospitalization following delivery longer than 7 days

Exclusion criteria for **infants** are as follows:

- Has major birth defect(s) including physical anomalies including limb malformations/absence of limbs or chromosomal abnormalities
- Diagnosed with neonatal encephalopathy, metabolic disorder, stroke, intracranial hemorrhage, or meningitis during birth hospitalization
- Received any major surgical intervention during the birth hospitalization or required a prolonged birth hospitalization (prolonged hospitalization being any hospitalization longer than 7 days)

## 5.3 Lifestyle Considerations

See inclusion/exclusion criteria, the study does not impose lifestyle considerations.

## 5.4 Screen Failures

We define screen failures as birthing parent/infant dyads that are screened for participation in the trial but do not meet all the criteria for participation. Screen failures also include birthing parent/infant dyads that meet the criteria for participation based on screening but choose not to participate in the study. Individuals who do not qualify after initial screening will not be rescreened for this pilot feasibility study.

## 5.5 Strategies for Recruitment and Retention

A multi-pronged approach will be used for recruitment at all 3 sites. Individuals will be recruited prepartum in the clinic setting as well as during the birth hospitalization. As it will be critical to ensure timely consenting/enrollment, recruitment and consenting metrics will be reviewed monthly. Consenting/enrollment will be competitive, with ideally, each site contributing an equal number of participants. However, as some sites will have more recruitment barriers, no one site will be allowed to contribute more than half of the total study goal number of dyads (each site cannot enroll more than 10 dyads). In order to reach the study enrollment goal over the 3-month period, we expect a monthly enrollment rate of at least 2-3 eligible dyads per site that go on to complete the first coaching session. If noted that the number consented is not reaching the monthly target number of participants, then alternative strategies will be implemented. Additionally, if there are concerns with the rate of recruitment, the DSMB will be contacted and notified of the concern, at which time the study team will provide possible solutions, and next steps will be discussed. All consenting will occur after delivery of the infant. Please see figure 3 for additional recruitment strategies based on the state cannabinoid legalization status of each study site.

Outpatient Recruitment

Within the clinic setting, flyers will be placed in outpatient obstetrical and family medicine clinics affiliated with each of the study sites for birthing parent self-referral. Additionally, providers will be notified of the study enrollment criteria and can distribute a flyer to any pregnant individual that may qualify to participate. Flyers will also be distributed to local cannabinoid dispensaries in the states where recreational use of cannabinoids is legal. If we screen an individual prenatally who is identified via self- or outpatient healthcare provider referral, the screening will be logged. This will ensure that an individual that declines study participation or does not qualify to participate will not be re-approached during the birth hospitalization. Additionally, social media will be considered as a possible recruitment strategy. Possible strategies for the use of social media will not involve potential participants uploading anything on social media sites that would indicate they may have used marijuana during pregnancy.

Inpatient Recruitment

Different options may be implemented by the local study team, which could include but are not restricted to those listed below. In all aspects of the study, the study personnel will follow local HIPAA regulations and remain mindful of privacy and confidentiality.

Birthing parents with a positive toxicology screen for THC during the birth hospitalization will be identified by an automated daily chart review of the electronic medical record (EMR) with the daily report of potentially eligible birthing parents automatically sent via secure email from the EMR to the local study team. A partial HIPAA waiver for this pre-screening procedure to review medical records to identity potential participants will be sought prior to study initiation. Individuals identified in this manner will then be approached by a study team member to discuss the study in more detail. Study team members will be trained to interact using a non-judgmental approach. Study personnel interacting with participants have completed institutional implicit bias training, which will aide in a non-judgmental approach. This approach will be reviewed and emphasized during the study training and consent process. This field will be queried to generate the daily EMR-generated report that will be reviewed daily by the study team to identify potential participants when they are admitted for the birth hospitalization. Potential participants referred by the clinical care team will be approached by study team members to introduce the study and confirm eligibility. Study team members are embedded within the clinical care team and will help providers identify potential participants prior to engagement. We will obtain a partial HIPAA waiver for this pre-screening procedure only; informed consent will be obtained prior to any data collection or other study involvement.


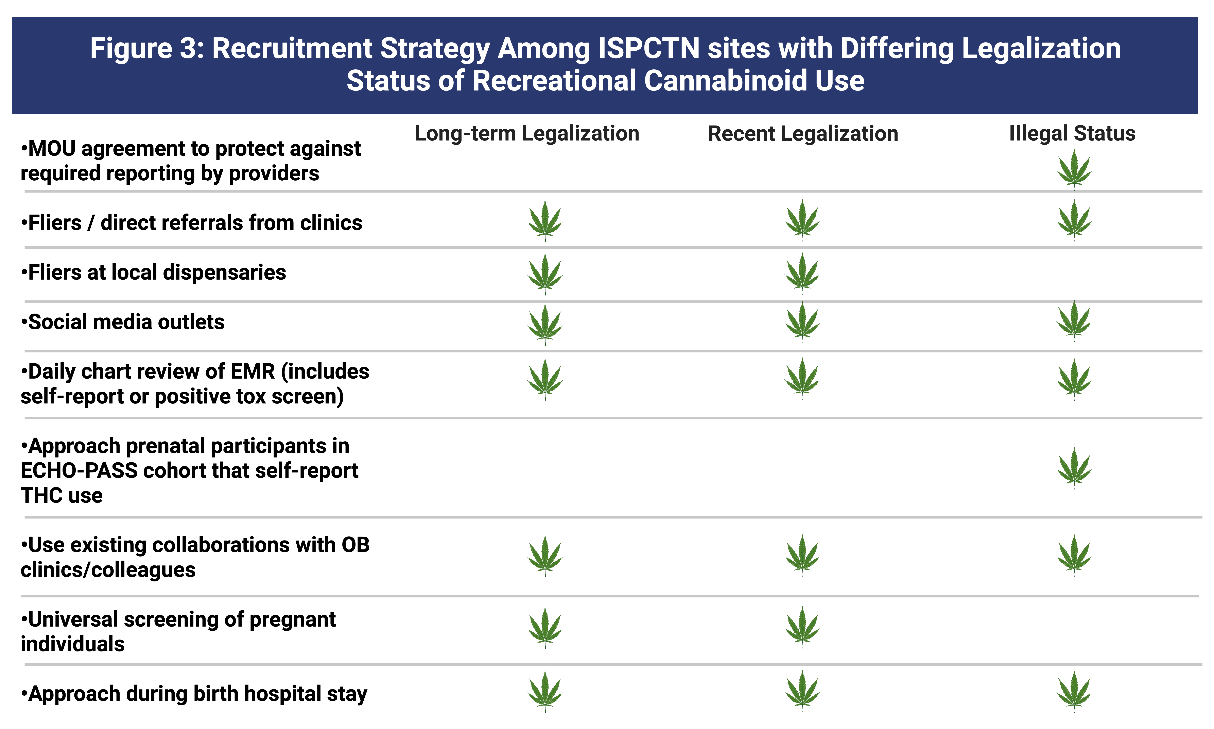


Tobacco use and/or vaping will not be an exclusion criterion due to the frequency of concurrent use in the proposed study population. Given the voluntary nature of the study and that consent relies on self-reported cannabinoid use, some potentially eligible individuals may not self-identify, which could limit the number consented. This aspect is one of the feasibility components that will be assessed with this pilot project.


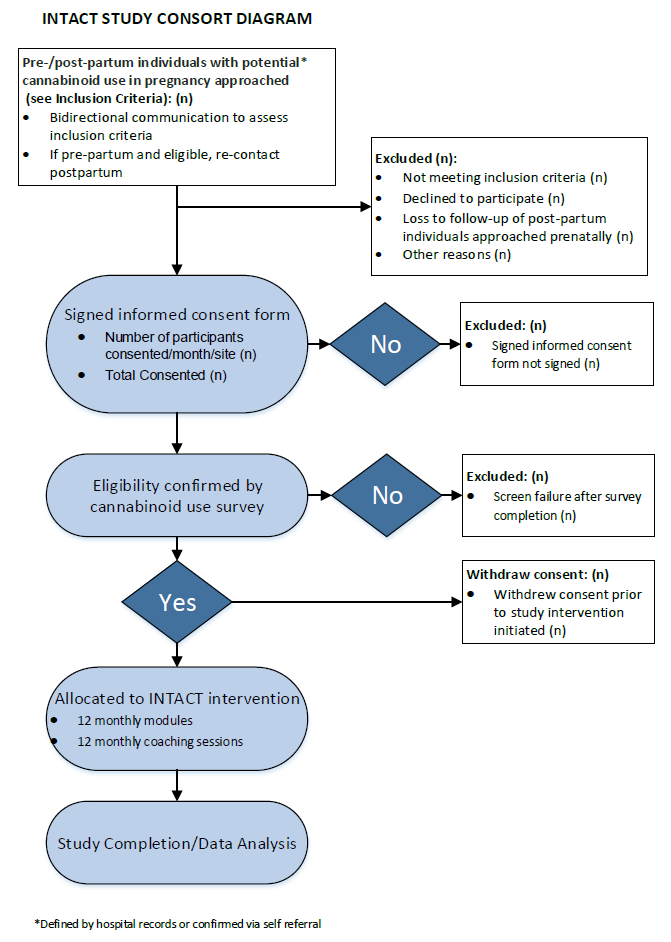
To ensure the study meets the network goal of providing medically underserved and rural populations with access to state-of-the-art clinical trials, we will also collect information on each birthing parent/infant dyad regarding diversity, equity, and inclusion. The zip code will be used to identify individuals from a rural area, defined by a rural-urban community area (RUCA) code of >4. Individuals will also be asked to self-identify as rural or non-rural. This will be monitored during enrollment in order to strive to enroll 30% of participants from rural areas.

At the time of recruitment, the NIH Certificate of Confidentiality will be discussed with potential participants. The CoC protects information, documents, and/or biospecimens that contain identifiable, sensitive information related to a participant. The CoC policy and 42 U.S. Code 241(d) define identifiable, sensitive information as information that is about an individual and that is gathered or used during the course of research, where an individual is identified or for which there is at least a very small risk, that some combination of the information, a request for the information, and other available data sources could be used to deduce the identity of an individual. Information that is protected by a CoC is immune from the legal process and is not admissible as evidence unless the participant consents to this disclosure.

Participant Retention

If individuals do not complete the monthly intervention tasks, the study team will contact them via their preferred contact method to determine if the birthing parent would like to continue in the study, and if so, provide a reminder of the outstanding tasks and assist with any technical or scheduling difficulties. This will occur if the birthing parent does not complete the monthly online module or the coaching session (See Section 7.3 for additional details).

Participants will be reimbursed for their time and effort to participate in the study. An internet stipend will be provided from months 1 through 12 to subsidize internet access to complete study activities. Additionally, an incentive will be provided upon completion of the study orientation. Each month the participant will receive a small incentive if they complete the INTACT interventions for that month, which consists of participating in the coaching session. This will be provided for months 1 through 12.

# 6 Study Intervention

## 6.1 Study Intervention(s) Administration

### 6.1.1 Study Intervention Description

The INTACT intervention includes contingent responding training completed via online modules monthly with personalized coaching provided to the birthing parent by a trained study team interventionist/coach at the monthly coaching session. Training will be provided to the study interventionist/coach to obtain certification in the ePALS (remote Play and Learning Strategies) methodology (interventionists will be research coordinators). Two research coordinators at each site (six individuals) will undergo ePALS certification training, which will require participation in 4 remote training sessions lasting 2 hours each, along with self-study. After training completion, each coordinator will only receive ePALS certification after passing a knowledge assessment test and practical exam for which the interventionist will submit videos of mock ePALS sessions to the University of Texas Health Science Center at Houston. After certification, the research coordinators will be the interventionists/coaches who will then conduct every monthly coaching session at his/her ISPCTN site. Some of the coaching sessions with the participant will be monitored by another interventionist to ensure fidelity throughout the pilot feasibility trial; the Consent and HIPAA forms reflect this quality control requirement.

After approaching, consenting, and confirming eligibility, birthing parent participants will be oriented to the intervention beginning with basic training. During this time, the birthing parent participant will be instructed on how to access the INTACT modules. Each month, a new module will be accessible by the birthing parent for the 12 months post enrollment and confirmation of eligibility. Birthing parent participants will also be instructed on how to schedule and attend the monthly virtual sessions with the interventionist/coach to receive individualized feedback and coaching using the web-based conferencing platform.

The birthing parent participant will complete an INTACT intervention module each month. A total of 11 modules will be available for completion by the end of the study. The INTACT intervention modules are adapted from the ePALS project. The content of the modules is the same as previously used and published by the ePALS group with different populations of interest. The timing of the modules (1 per month) and the population of interest are what is changed for this study. The modules were designed to facilitate change in caregivers’ behaviors across cognitive stimulation and emotional support domains.^48^ The monthly modules contain video segments to illustrate concepts and generate points of discussion for the follow-up coaching sessions. The skills taught in the modules include reading infant signals, responding with warm, sensitive behaviors, maintaining versus redirecting infants’ focus of attention, watching for opportunities to introduce an object or social game, stimulating language development by naming objects and action in combination with physical demonstrations, and incorporating the use of the constellation of behaviors during daily tasks such as dressing and feeding. The online modules are up to 60 minutes in length.

Monthly coaching sessions with the birthing parent participant and a trained study interventionist coach will occur via a cloud-based video conferencing service, lasting no more than 60 minutes. Each study site will have two trained interventionists/coaches that will have completed the adapted ePALS certification as described above. The interventionist/coach should have a bachelor’s degree but does not require additional education. The interventionist/coach may be a research coordinator or another individual identified at each site. During the monthly sessions, contingent caregiving will be discussed with a focus on trust, responsiveness, attachment, acceptance, accountability, and reflection. The following monthly session will begin with reflecting on the prior goal and achievements with the birthing parent before covering new contingent parenting techniques and feedback. Some of these monthly sessions are review sessions designed to consolidate the learning and the opportunity to demonstrate their new skills to another adult that participates in the baby’s life. During sessions the birthing parent participant may interact with the infant to model or practice behaviors with the interventionist/coach observing to provide immediate feedback. As there will be interaction with the infant during the sessions, the infants are expected to be present during the coaching session.

There will be a total of 12 sessions to be completed by each participant during the study period (including the introductory session), averaging 1 session per month. During the pilot feasibility trial, some sessions conducted by the site interventionist/coach will be monitored by another interventionist to ensure consistent training and feedback/coaching is provided to the birthing parent. If needed, an interventionist/coach may receive directed retraining if concerns arise during the monitored session. Each coaching session is expected to take no more than 60 minutes, with the time for all sessions being less than 12 hours for each participant over the course of 12 months. We will require a minimum of 2 weeks between each session to ensure the intervention continues throughout the study period, without clusters of sessions being completed in a short time to ensure there is steady dosing over the participant’s study period. The birthing parent participant is expected to complete the INTACT online module each month in-between the cloud-based video conferencing meetings/sessions, which takes no more than 60 minutes per module. Thus, the expected required birthing parent participant time devoted to study-related activities should not surpass 30 hours over the 12-month study enrollment period (accounting for consenting, survey completion, and initial basic training).

## 6.2 Preparation/Handling/Storage/Accountability

### 6.2.1 Acquisition and accountability

This study is a behavioral interventional trial which does not include any medication. Please see section 6.1 for a detailed review of the intervention.

### 6.2.2 Appearance, Packaging, and Labeling

This study does not include any devices with packaging or labeling.

## 6.3 Measures to Minimize Bias: Randomization and Blinding

This study is a behavioral intervention study to assess the feasibility of recruitment, retention, and completion of the intervention in this population. Therefore, there is no control group and no randomization. As there is no control group, no blinding will be completed.

## 6.4 Study Intervention Compliance

Compliance will be assessed based on the completion of the INTACT intervention, as discussed in Objective 3. This consists of: meeting with a trained interventionist/coach who can provide tailored feedback and advice to the participant focused on the specific aspect of contingent caregiving covered by that month’s online module.

# 7 Study Intervention Discontinuation and Participant Discontinuation/Withdrawal

## 7.1 Discontinuation of Study Intervention

The study intervention will only be discontinued if the participant chooses to withdraw from the study (see below) or if the birthing parent participant does not retain custody of the child throughout the study. If a birthing parent participant loses their device that they use to access study activities for a period of ≥3 consecutive coaching sessions, the study intervention will be discontinued.

## 7.2 Participant Discontinuation/Withdrawal from the Study

If the birthing parent participant does not retain custody of the child throughout the study, they will also be withdrawn from the study.

A birthing parent/infant dyad may withdraw from the trial at their own request, or the site investigator can withdraw a birthing parent/infant dyad. Reasons for withdrawal may include but are not limited to:

- Birthing parent/infant choose to withdraw at their own request
- Birthing parent/infant dyad is lost to follow-up
- Birthing parent/infant dyad is unwilling or becomes unable to comply with the study procedures required by the protocol
- Birthing parent loses custody of the infant

ISPCTN site personnel will record the reasons for withdrawal in the trial documentation. Sites can replace the birthing parent/infant dyad in the case of a screen failure, including those that are deemed not eligible after obtaining informed consent and completing the cannabinoid use survey. Sites can also replace the birthing parent/infant dyad if they withdraw from the study before beginning the first month’s coaching session.

## 7.3 Lost to Follow-Up

A participant is considered lost to follow-up when there has been no contact for three consecutive coaching sessions despite maximum attempts. The site research team must make reasonable attempts to contact the participant using resources such as phone, text, email, private messaging through social media, and medical records, as permitted by the central IRB. If the coordinator is able to reach a self-identifying voicemail or responder, a reasonable attempt at contact may be defined as two texts or phone calls during working hours, one in the evening and one on the weekend. Research coordinators will keep a log of all methods utilized. Once a participant is lost to follow-up, the site research team may stop contacting them.

Participants will be asked if they are willing to provide names and contact information of back-up contact(s) or other ways for the research team to stay in contact throughout the year-long study. Participants will be informed that once the participant provides back-up/other contact information, the research team may use those method(s) to contact the participant, and that back-up contacts may then learn that the participant is part of this study.

# 8 Study Assessments and Procedures

## 8.1 Efficacy Assessments

**PRIMARY OUTCOME MEASURES**

**Objective 1 – Participant Recruitment.** All birthing parents approached by the study team for study participation will be tracked to determine the number that are approached, consented, and confirmed to be eligible for study participation. We aim to consent and confirm eligibility in at least 2-3 individuals at each site per month, with enrollment duration lasting 3 months, averaging at least 6.67 individuals per month over all sites. We will have a log of participants consented, site location, and the date. This will allow the number of individuals consented per month to be calculated. For a future randomized controlled trial, we would need to consent and maintain eligibility in at least 360 participants in total, or 20 per site in each of the 18 ISPCTN sites. This sample size will provide statistical power of 0.83 to detect a mean difference of 5 scale points between the intervention and comparison conditions on the Bayley Scales of Infant and Toddler Development, Fourth Edition, which would be the primary outcome measure. A difference of 5 scale points on the Bayley-IV is one-third of a standard deviation which equates to an effect size *d* = 0.33, which is between the thresholds for a small and moderate effect (0.20 and 0.50, respectively). The sample size estimation assumes an intra-class correlation of 0.05 within ISPCTN sites. Thus, consenting and confirming eligibility of at least 2-3 individuals per site per month in the feasibility trial will ensure that consenting and confirming eligibility in 2 individuals per site per month in a randomized controlled trial is feasible.

The proportion of birthing parent participants recruited is calculated as the number of birthing parents that are approached, consented, and determined to meet study eligibility divided by the number of birthing parents approached for participation. The number of individuals approached by a study team member will be recorded to know the proportion that consent after being approached. An approached birthing parent is defined as having a study team member discussing the trial with a possible unique participant (if the study team member discusses the trial with the same possible participant more than once, this will be documented as only 1 individual approached) with bi-directional communication. We will calculate the consented proportion to total individuals approached to inform the number of individuals that would need to be approached for a larger clinical trial. We aim to have at least 20% of approached individuals consent and be determined to be eligible for study participation.

**Objective 2 – Participant Completion.** We will calculate participant completion as the percent of birthing parent /infant dyads that complete the final monthly coaching session when the child reaches 12 months of age. The numerator will be the number of participants that complete the final monthly coaching session when the child is 12 months of age, and the denominator will be the total number of participants that were approached, consented, and determined to be eligible. We will then calculate the completion rate for each site and across all sites, with at least 70% of dyads completing the final coaching session across all sites as the goal.

**Objective 3 – Participant Adherence.** We will calculate the number of participants who receive at least 70% of the number of INTACT intervention coaching sessions. This will be done by taking the number of INTACT intervention coaching sessions completed by the study participants that were approached, consented, and met eligibility for participation. The total number of monthly coaching sessions possible will serve as the denominator (12 per dyad), and the total number of monthly coaching sessions completed by the birthing parent/infant dyad will serve as the numerator. We will calculate this ratio for each birthing parent/infant dyad and aim to have 80% of the participants complete a sufficient number of INTACT intervention coaching sessions completed, defined as at least 8 coaching sessions (70% of the total number of INTACT intervention coaching sessions possible).

## 8.2 Safety and Other Assessments

Other measures for this trial include the Cannabinoid Use Survey. Because this is a study of a behavioral intervention, related adverse events (AEs) and serious adverse events (SAEs) are not expected. While the study team will not solicit AEs or SAEs, given the minimal biological/medical risk of the study, the study will provide all participants with a telephone number to report AEs/SAEs. All reported AEs will be evaluated for severity, action taken, seriousness, outcome, and relationship to the study intervention. The study team will track and record all AEs that are severe, serious, and/or related to the study intervention. AEs and SAEs will be collected for both the parent and infant on this study.

## 8.3 Adverse Events and Serious Adverse Events

### 8.3.1 Definition of Adverse Events (AE)

An AE is defined as any untoward medical occurrence associated with the use of an intervention in humans, whether or not considered intervention related.

### 8.3.2 Definition of Serious Adverse Events (SAE)

We will consider an AE or suspected adverse reaction to be “serious” if, in the view of the PIs, medical monitor, or DSMB, it results in any of the following outcomes:

- Death
- Life-threatening AE
- Inpatient hospitalization or prolongation of existing hospitalization
- Persistent or significant incapacitation or substantial disruption of the ability to conduct normal life functions
- Congenital anomaly/birth defect

Important medical events that may not result in death, be life-threatening, or require hospitalization may be considered serious when, based upon appropriate medical judgment, they may jeopardize the participant and may require medical or surgical intervention to prevent one of the outcomes listed in the SAE definition.

### 8.3.3 Classification of an Adverse Event

#### 8.3.3.1 Severity of Event

The site research team will use the following guidelines to describe severity. The site investigator will determine severity.

- **Mild:** Event requires minimal or no treatment and does not interfere with the participant’s daily activities.
- **Moderate:** Event results in a low level of inconvenience or concern with the therapeutic measure. Moderate events may cause some interference with functioning.
- **Severe:** Event interrupts a participant’s usual daily activity and may require systemic drug therapy or other treatment. Severe events are usually potentially life-threatening or incapacitating. Of note, the term “severe” does not necessarily equate to “serious.”

#### 8.3.3.2 Relationship to Study Intervention

The site research team will grade the degree of certainty about causality by using the following categories:

- **Related:** The AE is known to occur with the study procedures, there is a reasonable possibility that the study procedure caused the AE, or there is a temporal relationship between the study procedures and the event. Reasonable possibility means that there is evidence to suggest a causal relationship between the study procedures and the AE.
- **Not Related:** There is not a reasonable possibility that the study procedures caused the event, there is no temporal relationship between the study procedures and the event onset, or an alternate etiology has been established.

#### 8.3.3.3 Expectedness

The site investigator or qualified clinical designee will be responsible for determining whether a severe AE or SAE is expected or unexpected. A severe AE or SAE will be considered unexpected if the nature, severity, or frequency of the event is not consistent with risk of the underlying chronic medical condition, or is not an event measured in study data collection (hospitalization, ED visit etc.), or is not related to function of the study intervention.

Potential expected event could include the following:

- Common illnesses and infections associated with the following organ classes:  Respiratory, Gastrointestinal, Skin, Eyes, Ears, etc.
- Developmental delay
- Slow/poor weight gain
- Need for emergency room visits secondary to physical injuries (including but not limited to falls, concern for fractures or nursemaid's elbow)
- Various neurological disorders and/or deficiencies

### 8.3.4 Time Period and Frequency for Event Assessment and Follow-Up

For this study, the protocol study team will collect all severe AEs, SAEs, and AEs related to the study procedures. The occurrence of a SAE may come to the attention of study personnel during the hospital stay, by the clinical team with administration of questionnaires, or by the medical monitor upon reviewing data. The site research team will capture all AEs on the appropriate case report form following the instructions and timelines provided in the MOP. Information to be collected includes event description, date/time of onset, date/time of resolution, clinician’s assessment of severity, relationship to study intervention and time of resolution/stabilization of the event. Site research teams must follow all severe and SAEs until there is resolution, the condition stabilizes, the event is otherwise explained or judged by the site PI or a qualified clinician designee to be no longer clinically significant, or the participant is lost to follow-up.

Any medical condition that is present at the time the participant is screened will be considered as baseline and not reported as an AE. However, at the onset of the intervention and anytime during the study, if the study participants condition deteriorates and meets the definition of a severe AE or SAE, it will be recorded as an AE. Changes in the severity of an AE will be documented to allow an assessment of the duration of the event at each level of severity to be performed.

### 8.3.5 Adverse Event Reporting

Site coordinators and/or the study team will record all severe and related AEs in the electronic data capture (EDC) system, include them in the statistical analysis, and follow the reporting procedures outlined in the trial-specific MOP. The MOP section on reporting severe AEs will encompass the requirements of:

1. Reviewing IRB’s policies and procedures
2. Standard Operating Procedures (SOPs) for the Streamlined, Multisite, Accelerated Resources for Trials IRB Reliance platform (SMART IRB)
3. ICH E6(R2), GCP: Integrated Addendum to ICH E6(R1): Guidance for Industry
4. Following local IRB’s policies and procedures, when applicable. If there are discrepancies between the procedures, we will follow the most stringent of the procedures.

If there are discrepancies between the procedures, we will follow the most stringent of the requirements.

The DCOC will report severe and related AEs to the cIRB for its yearly continuing review. The DCOC will report to the Central IRB (cIRB), as specified in the study-specific IRB communication plan, which is based on recommendations from the SMART IRB agreement. Sites will also report severe AEs to the local IRB, per local IRB policies and procedures, and make them available to the sponsor (DCOC) on a continuing basis through the EDC system. The study team/sponsor will report to the DSMB, per the DSMB charter.

The trial-specific MOP will describe the details of the reporting structure and additional details related to timelines for reporting.

### 8.3.6 Serious Adverse Event Reporting

The clinical coordinator will record all SAEs in the EDC and the DCOC will report SAEs. The DCOC will notify monitoring personnel (e.g., medical monitor) and monitoring bodies (e.g., DSMB), per the DSMB charter. The DCOC will make these reports whether or not the trial clinician or medical monitor consider the event trial-intervention related. Reports must include an assessment of whether there is a reasonable possibility that the trial intervention caused the event. Reports to the reviewing IRB will be made yearly for continuing review. The reports to the local IRB will be made per local IRB policies and procedures. Reports to the DCOC will be made immediately, within 48 hours of finding out about the event.

Study personnel will follow all SAEs until satisfactorily resolved or the ISPCTN site investigator deems the event is chronic, or the participant is stable.

### 8.3.7 Reporting Events to Participants

We will notify participants of those trial-related (or potentially trial-related) SAEs that may affect their willingness to continue with the trial or the future health of the mother or the infant. Any of the following can determine if study personnel should contact participants: the IRB, the medical monitor, the DSMB, or the PIs. The person or oversight body that makes the determination will inform the DCOC, which will instruct the ISPCTN site investigators and coordinators to contact the participants enrolled through their site.

ISPCTN site research staff will record any contact with participants, if necessary, in the EDC and/or trial log.

### 8.3.8 Events of Special Interest

We have not identified other reportable events for this trial.

### 8.3.9 Reporting of Pregnancy

We will not report pregnancies for this trial.

## 8.4 Unanticipated Problems

### 8.4.1 Definition of Unanticipated Problems (UP)

The Office for Human Research Protections considers unanticipated problems (UPs) involving risks to participants or others to include, in general, any incident, experience, or outcome that meets all the following criteria:

- Unexpected in terms of nature, severity, or frequency given 1) the research procedures that are described in the protocol-related documents, such as the IRB-approved research protocol and informed consent document; and 2) the characteristics of the participant population being studied; AND
- Related or possibly related to participation in the research (“possibly related” means there is a reasonable possibility that the incident, experience, or outcome may have been caused by the procedures involved in the research); AND
- Suggests that the research placed participants or others at a greater risk of harm (including physical, psychological, economic, or social harm) than study personnel previously knew or recognized.

An UP is not necessarily an AE or an SAE, but study personnel must report these. For example, a breach of confidentiality is an example of an UP that is not an AE or SAE. The UP report will include the following:

- Protocol identification information
- A detailed description of the event, incident, experience, or outcome
- An explanation of the basis for determining that the event, incident, experience, or outcome represents an UP
- A description of any changes to the protocol or other corrective actions that have been taken or are proposed in response to the UP

### 8.4.2 Unanticipated Problem Reporting

Study personnel must report UPs to the reviewing IRB, via the method(s) specified in the MOP, and directly to the DCOC. The DCOC will notify the PIs and the DSMB. Other persons or groups will receive reports as specified in the DSMB charter. Study personnel must report these UPs to the reviewing IRB according to its policies and procedures and report to local institutions according to the rules and regulations of the local institution. An unexpected death or life-threatening event will be reported immediately to the IRB office or IRB chair. All other unexpected events will be reported within 10 days of the event or notification of the event if non-local.

### 8.4.3 Reporting Unanticipated Problems to Participants

For reporting UPs to participants, we will follow the same procedures as described for reporting events to participants.

# 9 Statistical Considerations

## 9.1 Statistical Hypotheses

| **Objectives:** | \| **Primary Objective:** Conduct a rigorous feasibility pilot study of a novel program to improve neurodevelopmental outcomes in infants prenatally exposed to cannabinoids as the basis for a future randomized controlled trial. We will conduct the feasibility trial in three ISPCTN sites to assess the following variables: participant recruitment, participant completion when the infant is 12 months of age, and completion of overall total number of INTACT intervention coaching sessions. This information will inform a future randomized controlled trial, at which time a clinical outcome assessment with the Bayley Scales of Infant and Toddler Development Screening Test, 4^th^ Edition (Bayley-IV) will be completed at 2 years of age. Thus, no clinical outcome assessment will be completed in this feasibility pilot study.  **Objective 1: Participant Recruitment:** To examine if birthing parents can be approached, consented, and determined to be eligible in the study utilizing multi-faceted recruitment approaches, which may include distributing flyers in the obstetrical and family medicine clinics (for individuals with self-report) and utilizing electronic health record (EHR) review to identify and approach pregnant and post-partum individuals with positive toxicology screen(s). Providers may give flyers individually to birthing parents in the obstetrical and family medicine clinics if the individual uses cannabinoids in pregnancy. All birthing parents approached by the study team for study participation will be tracked. **Hypothesis:** The percentage of participants approached, consented, and determined to be eligible for study participation will be greater than 20% of those approached, consistent with other similar feasibility studies.  **Objective 2: Participant Completion:** To evaluate participant completion, as measured by the percent of birthing parent/infant dyads that complete the final coaching session scheduled when the child is 12 months of age (recruitment goal of n=20 dyads) compared to the number of individuals approached, consented, and determined to be eligible. **Hypothesis:** This study will have participant completion of at least 70% (n≥14) at the final visit when the child is 12 months of age.  **Objective 3: Participant Adherence:** To evaluate the number of individual INTACT intervention coaching sessions completed. This will be measured by the total number of sessions completed compared to the total number of possible sessions (n=12 sessions) for the individuals approached, consented, and determined to be eligible for study participation. **Hypothesis**: 80% (n≥16) of the participants will receive a sufficient number of INTACT intervention coaching sessions completed, defined as at least 8 of the 12 sessions.^1^ \| \| --- \| |
| --- | --- | --- |
| **Endpoints:** | \| Primary Endpoints:   1. Whether a potential participant approached for study participation will eventually be consented and determined to be eligible for study participation. 2. Whether a birthing parent/infant dyad will complete the final coaching session when the child is 12 months of age. 3. Whether a birthing parent/infant dyad will complete the sufficient number of INTACT intervention coaching sessions, defined as completion of at least 8 out of 12 coaching sessions. \|  \| \| --- \| --- \| |

## 9.2 Sample Size Determination

In **Objective 1,** we anticipate 20 eligible dyads will be approached, consented, and determined to be eligible for study participation, yielding a targeted rate of recruitment of at least 20% of the approached population. A null hypothesis of a rate of 10% will be rejected with 0.89 power using a one-sided exact test at 0.05 significance level.

The intent of **Objective 2** is to determine if the retention rate is ≥70%. We anticipate at least 14 dyads will complete the final coaching session for a retention rate of 70% or above. With 20 participants, the null hypothesis that the retention rate is 40% can be tested against the alternative that it is 70% at the one-sided 0.05 significance level with a power of 0.88. With 20 participants, the null hypothesis will be rejected if the number of participants who are retained is ≥14.

The intent of **Objective 3** is to determine if the participant adherence rate is ≥80%. We anticipate at least 16 out of 20 birthing parent/infant dyad will complete the required number of INTACT intervention coaching sessions. With 20 participants, the null hypothesis that the adherence rate is 50% can be tested against the alternative that it is 80% at the one-sided 0.05 significance level with a power of 0.90. With 20 participants, the null hypothesis will be rejected if the number of participants who are adherent is ≥16.

## 9.3 Populations for Analyses

The population for analysis for objective 1 is that which is approached for study participation. The population for analysis for objectives 2 and 3 is consented dyads with confirmed eligibility.

## 9.4 Statistical Analyses

### 9.4.1 General Approach

Following finalization of the protocol, but prior to data lock, the DCOC statistical team will issue a Statistical Analysis Plan (SAP) as a separate document, which will provide detailed analytical plans for the set of analyses outlined below. The statistical team will conduct all statistical analyses following the statistical principles for clinical trials as specified in ICH Statistical Principles for Clinical Trials (ICH Topic E9). The study team will describe and justify any deviations from the planned analyses in the final integrated clinical study report. The study team will present all study data and summary tables for the overall study and by study sites.

**Descriptive Statistics:** At baseline, demographics and cannabinoid usage based on the Cannabinoid Use Survey will be collected. All numerical variables will be summarized using mean ± standard deviation and median (minimum, maximum). All categorical variables will be summarized using frequency (in %).

**Statistical Inference:** Considering the small sample size proposed in the pilot study, exact methods will be used in statistical inference. Hypothesis tests will be one-sided with a significance level of 0.05.

### 9.4.2 Analysis of the Primary Efficacy Endpoint(s)

**Objective 1: Participant Recruitment.**

The primary endpoint of recruitment will be summarized using frequency (in %). A one-sided exact Clopper-Pearson (or exact) test will be used to test against a null rate of 10%.

**Objective 2: Participant Completion.**

The endpoint of study completion of required study assessments (or participant completion) will be summarized using frequency (in %). A one-sided exact Clopper-Pearson (or exact) test will be used to test against a null rate of 40%.

**Objective 3: Participant Adherence.**

The endpoint of completion of a sufficient number of INTACT intervention coaching sessions (or participant adherence) completed will be summarized using frequency (in %). A one-sided exact Clopper-Pearson test will be used to test against a null rate of 50%.

### 9.4.4 Safety Analyses

We will detail all severe AEs and SAEs experienced by the birthing parent/infant dyad. The DCOC will present summary statistics overall and by individual site.

### 9.4.5 Baseline Descriptive Statistics

Baseline characteristics will be summarized using descriptive statistics specified in Section 9.4.1.

### 9.4.8 Tabulation of Individual Participant Data

None

# 10 Supporting Documentation and Operational Considerations

## 10.1 Regulatory, Ethical, and Study Oversight Considerations

### 10.1.1 Informed Consent Process

To meet study objectives, we will consent up to 50 participant dyads. Birthing parent participants will then complete the Cannabinoid Use Survey for the final eligibility screen. Up to 20 eligible participant dyads will be assigned to the intervention, not including participants that withdraw from the study before beginning the first month’s coaching session. Recruitment will continue until one of these targets has been met (consent or intervention assignment).

#### 10.1.1.1 Consent/assent and Other Informational Documents Provided to participants

ISPCTN coordinators or research-trained designees will give participants consent forms describing in detail the trial intervention, trial procedures (including that study personnel will access participants’ medical records and alternative contact information), and risks. We will require written documentation of the informed consent prior to completion of the Cannabinoid Use Survey and prior to starting or administering the intervention.

#### 10.1.1.2 Consent Procedures and Documentation

Informed consent is a process that starts before a participant agrees to participate in the trial and continues throughout the individual’s trial participation. For this trial, the participants include birthing parent plus infant dyads. Site coordinators will inform birthing parents who are approached that participation is voluntary, that they may withdraw from the trial at any time without prejudice, and that nonparticipation will not adversely affect their child’s or their own medical care.

Site coordinators or other research team members will contact potential participants to describe the study. Flyers will have a phone number to call to discuss potential participation with a site coordinator. During screening, the research team member will confirm that the potential participant meets all inclusion criteria and none of the exclusion criteria. Once potential participants agree to participate in the study, the research team member will again confirm the potential participant remains eligible. If so, the research team member will conduct the informed consent process with participants. The study team will collect informed written consent and Health Insurance Portability and Accountability Act (HIPAA) authorization. Site research teams can conduct the informed consent process in person or remotely via HIPAA-compliant means (e.g., via telephone, REDCap, or video conference platform, etc.). Sites must complete informed consent procedures, including obtaining a signed informed consent form and HIPAA authorization for research form before enrollment or intervention procedures can begin. Sites must also return a signed copy of all consent and HIPAA authorization forms to the participants. Research teams must document the informed consent process, following the current UAMS Institutional Review Board policy.

Site research staff will ask participants to read and review the consent form and HIPPA authorization document(s). Site research staff will explain the research trial to the participant in terms the participant can understand and answer any questions that may arise. The explanation will state the purposes, procedures, and potential risks of the trial and describe participants’ rights as research participants. Participants will have the opportunity to carefully review the written consent form and ask questions before signing. The site research staff will give participants the opportunity to discuss the trial with their family or surrogates or think about participating in the trial prior to agreeing to participate. Consenting procedures will conform to the site’s and UAMS central IRB’s approved consenting procedures. These may include any of the following to indicate consent to the study:

- Sign the form and hand it back to site research staff;
- Sign the form and postal mail it back to site research staff;
- Print, sign, scan, and electronically or fax return to site research staff;
- Print, sign, photograph the signature page, and text or email a picture of the signature page to site research staff;
- Type the signature and date into the electronic version of the consent document, save, and electronically return to the site research staff with a note stating that this is the legally authorized representative’s electronic signature. If this method is used, site research staff will confirm the returned consent forms containing the same text as the consent forms sent to the participant;
- Use certified e-signature software to sign the consent document, save, and return the document to the site research staff;
- Ask the participant to send a well-worded email back to the site that states the name of the study, that the participant has read and discussed the consent documents, and that the participant gives consent to participate.

When sites receive the participant’s signed consent form, the research team member who participated in the consent discussion will countersign the consent form or well-worded email and return a copy of the countersigned form or email to the participant. Site coordinators will ask participants to keep a copy of the consent form that is signed by all parties. Once the site and participants complete this process, the study team will consider the participant enrolled in the study. Due to the age (<2 years old) of the children participating in this study, assent will not be obtained.

If needed, remote consenting may be used to enroll a participant after the child has left the hospital. All communications will be done via HIPAA-compliant methods such as telephone, personal delivery of documents, US postal service, REDCap or other compliant electronic platform. The remote consent process will parallel the consent processed used for in-person consenting. The only difference will be the method(s) of communication. The study team will ensure that, as with in-person consenting, the parent/legal guardian of the participant is given sufficient opportunity to ask questions, is able to understand the nature of this study and what participation entails, and is provided a copy of the final, completed consent signed by all parties involved, including the research team member who obtained consent and, when applicable, the site investigator. This final, signed consent will be provided via a HIPAA-compliant method or a method that the parent/legal guardian of the participant has agreed to in writing. The site research team members working on the consenting process will ensure that any parent/legal guardian who is consenting remotely has the authority to consent for the child.

### 10.1.2 Study Discontinuation and Closure

This trial may also be suspended or stopped per any stopping/suspension indications, per NIH decision. Early termination may be permanent if there is sufficient cause.

The suspending or terminating party will provide, directly or indirectly, written notification documenting the reason for trial suspension or termination to the following, as applicable: protocol chairs, reviewing IRB, and the DCOC. DCOC would be responsible for communications within the ISPCTN. Persons and offices notified will include those specified, the reviewing IRB’s policies and procedures, and the SMART IRB policies and procedures.

The study personnel at each site will also contact trial participants and inform them of any changes to the trial visit schedule, including termination of study activities and any actions needed due to termination of study activities. Circumstances that may warrant termination or suspension include, but are not limited to:

- Determination of unexpected, significant, or unacceptable risk to participants
- Demonstration of efficacy that would warrant stopping
- Insufficient compliance to protocol requirements
- Data that are not sufficiently complete and/or evaluable
- Determination that all endpoints have been met
- Determination of futility

If the trial is temporarily suspended, it may resume once concerns about safety, protocol compliance, and data quality are addressed and are satisfactory to the DSMB, the reviewing IRB, the local IRBs (when applicable), the funding agency, and the sponsor (DCOC).

### 10.1.3 Confidentiality and Privacy

Sites and study team members will conduct all trial activities in as private a manner as possible.

Records will be maintained as required by the privacy and security rules promulgated by the Health Insurance Portability and Accountability Act (HIPAA, Title 45 of the CFR Part 164).

During the trial, site investigators and/or site coordinators will keep all trial records in secure locations that only authorized personnel can access. Examples of secure locations include but are not limited to: 1) locked file cabinet(s) in a limited (badge or key) access room, 2) password-protected computer systems. Study personnel may only transmit records that contain PHI, as defined by HIPAA, through an open email system if the personnel encrypt the data. Password protection alone is insufficient for data transmission through an open email system. After trial completion, access to trial records will be limited.

Certain bodies/institutions may need to review information, including the participant information, for any of the following reasons: to process information or to ensure compliance with the protocol and other applicable requirements (such as the policies and procedures of the cIRB). Institutions/bodies that may have access to the participants’ information include:

- UAMS IRB (cIRB) and other oversite offices
- IRB for the site through which the participant is consented
- OHRP
- DCOC
- NIH

Individuals with access to trial records will be:

- PI
- Site PIs / Co-Investigators
- Site coordinators
- Data managers at participants site(s)

To ensure privacy and confidentiality, a participant’s study data will be coded using a unique subject identifier assigned to them when they enter this study. This identifier is used to associate the participant with their data throughout the study, but does not rely on personal identifiable information (PII). The link between the participant and the subject identifier (or “key”) is securely stored and managed at the site according to their institutional standards. Once data analysis is complete, the study will be closed at the site level, severing the link between study data and PII. Data will be de-identified before sharing externally; data sent from the individual sites to the DCOC will be coded information and have no identifiers.

### 10.1.4 Multi-site Communications (IRB-related)

This study will be conducted at three sites within the ISCPTN network. All sites will cede to the UAMS IRB as the reviewing IRB (per SMART IRB definitions). The study-specific IRB-related communications plan was constructed from the SMART IRB template and uses SMART IRB recommendations for communications. This plan will be submitted to the IRB as a separate study-specific document. The DCOC will serve as the lead study team and will be the intermediary between the sites and the UAMS IRB as the central (or single) IRB (i.e., cIRB). Other types of communications (i.e., related to data, study deviations, etc.) between DCOC and the sites are detailed in their respective appropriate sections of this protocol.

### 10.1.5 Future Use of Stored Specimens and Data

No specimens will be collected or stored for this trial. Regarding stored data, site investigators, site coordinators, and study team members will document all trial interactions, and these will be password-protected in secured facilities/locations.

The study team will place participants’ de-identified data into one or more centralized database(s). The study team will share this data in compliance with the NIH data-sharing policy.

For future studies using any procedures or analyses not specified in this protocol, IRB approval is required.

### 10.1.6 Key Roles and Study Governance

### 10.1.7 Safety Oversight

Ensuring participant safety is the responsibility of all study team members, especially the PIs, site investigators, site coordinators, and monitors. A medical monitor and the DSMB will provide oversight.

The medical monitor will be a pediatrician with relevant expertise and will be independent of the trial. The NIH will convene the DSMB, and it will meet regularly, according to its charter.

The entities that will receive reports include but are not limited to, the DCOC and the NIH. The DSMB charter will provide additional information.

### 10.1.8 Clinical Monitoring

We will conduct clinical site monitoring to ensure that we protect the rights and well-being of study participants, that the reported trial data are accurate, complete, and verifiable, and that the conduct of the study complies with the currently approved protocol/amendment(s), with International Council for Harmonisation Good Clinical Practice, and with applicable regulatory requirements (for details specific to Protocol Adherence see Section 4.2.5).

- A member of the DCOC clinical operations staff or their designee will monitor the study.
- The clinical monitoring team will plan and conduct an on-site visit at least once during the course of the study and more often if needed for cause.
- Details of clinical site monitoring are in the Clinical Monitoring Plan, which will be included in the MOP. The plan describes who will conduct the monitoring, at what frequency monitoring will occur, at what level of detail monitoring will be performed, and how monitoring reports will be distributed.

### 10.1.9 Quality Assurance and Quality Control

Each IRB-approved site entering data will perform internal quality management of study conduct, data collection, documentation, and completion. Each site will follow the trial-specific MOP and any additional written site‑specific SOPs. The operating procedures will include, but are not limited to, procedures for: 1) performing the consent process; 2) data collection, entry, review, and submission processes; 3) assigning roles and responsibilities of site personnel; and 4) training methods for study staff. Sites will provide direct access to all their facilities, source data/documents, and reports for the purpose of monitoring and auditing by the DCOC and inspection by local and regulatory authorities. When electronic health records are source data/documents, sites must provide read-only access for the monitors and auditors, and anyone else authorized to inspect or verify records.

Following the DCOC monitoring SOPs, the monitors will verify that the clinical trial is conducted, and that data are generated, documented (recorded), and reported in compliance with the protocol, the trial‑specific Site Performance Plan, site-specific SOPs, the ICH GCP E6(R2), and applicable regulatory requirements.

The DCOC will implement quality control procedures for the database and DCOC-maintained records in accordance with the Site Performance Plan, MOP, data safety monitoring plan (DSMP), and applicable SOPs. The DCOC may communicate information about any data anomalies to the site(s) for clarification/resolution.

The DCOC will address issues uncovered during quality assurance, quality control, or monitoring activities through simple corrections or root-cause analysis, followed by instituting corrective and preventative action (CAPA), as appropriate and as described in the MOP.

### 10.1.10 Data Handling and Record Keeping

#### 10.1.10.1 Data Collection and Management Responsibilities

A formal data management plan will describe and document the data and workflow for the trial. The data management plan and associated documentation will specify all operations performed on data from origination to database lock, including detailed descriptions of source documentation, case report forms (CRF), instructions for completing forms, data handling and record-keeping procedures, procedures for data monitoring, and reconciliation procedures and coding dictionaries to be used, if applicable. The data management plan will also describe the specific data collection and management responsibilities required of the sponsor (DCOC), PIs, site investigators, site awardees, clinics, and DCOC. The data management plan contents will be consistent with those described in the Good Clinical Data Management Practices (GCDMP). The DCOC will provide the data management plan components that document operations performed on the data to the PIs for review and approval prior to implementation.

Data collection is the responsibility of the trial staff at the individual sites under the supervision of the site investigator. The site investigator is responsible for ensuring the accuracy, completeness, legibility, and timeliness of the data reported. All source documents must be completed using standard good documentation practices (i.e., the ALCOA-C method [attributable, legible, contemporaneous, original, accurate, and complete]).

It is best practice for site coordinators to use hardcopies of any data recorded on paper case-report forms or trial visit worksheets/assessment forms as source document worksheets for recording data for each participant consented. Data recorded in EDC derived from source documents must be consistent with the data recorded on the source documents.

Site personnel will enter data (including demographics and intervention-specific questionnaires) into an EDC system that complies with HIPAA regulations, provided by the DCOC. The EDC system includes password protection and internal quality checks, such as automatic range checks, to identify data that appear inconsistent, incomplete, or inaccurate. Study personnel will enter clinical data directly from the source documents. Data will be de-identified before sharing externally; data sent from the individual sites to the DCOC will be coded information and have no identifiers.

#### 10.1.10.2 Study Records Retention

Throughout the course of the trial, all sites will retain the source documents on site in accordance with current site-specific medical record storage procedures.

Sites must retain all trial documents in accordance with local and/or federal regulations, whichever is most stringent. Sites will not destroy any records without the written consent of the DCOC. The DCOC will inform all site investigators when they no longer need to retain these documents.

### 10.1.11 Protocol Deviations

Protocol deviations are any instances in which study team members and site personnel (e.g., site investigator, site coordinator, etc.) do not follow the study procedures as written in the protocol. Protocol deviations are not allowed unless the DCOC or Operational PI gives specific written permission for the deviation. Anyone who receives written permission for a protocol deviation must keep this with other study documentation. Sites must record all deviations in the trial source documents. Whenever a deviation occurs, the DCOC will conduct an assessment (which they do not need to write) of the severity and risk of the deviation. Depending on the results of the assessment, the DCOC will request/ensure that there is either a corrective action or a simple one-time correction, as appropriate. A corrective action institutes a process designed to keep the specific problem from reoccurring. Typically, assessors will determine the root cause of the problem to develop the most appropriate corrective action plan.

We will provide the specific methods for handling deviations in the trial-specific MOP and/or trial-specific SOPs.

### 10.1.12 Publication and Data Sharing Policy

We will conduct this trial in accordance with the following publication and data-sharing policies and regulations:

- **NIH Public Access Policy**, which ensures that the public has access to the published results of NIH-funded research. It requires scientists to submit final peer-reviewed journal manuscripts that arise from NIH funds to the digital archive PubMed Central upon acceptance for publication.
- **ECHO ISPCTN Publications and Presentations Policy**, which ensures accurate, responsible, and efficient communication of findings from ECHO ISPCTN clinical trials. The ECHO ISPCTN Steering Committee has approved and ratified the ECHO ISPCTN Publications and Presentations Policy, which includes representatives from all site awardees, as well as representatives from the NIH and the DCOC.

**NIH Data Sharing Policy and the policy** **on the Dissemination of NIH-Funded Clinical Trial Information and the Clinical Trials Registration and Results Information Submission Rule.** We will register this trial at ClinicalTrials.gov, and we will submit trial results to ClinicalTrials.gov. In addition, we will make every attempt to publish results in peer‑reviewed journals. Other researchers may request data from this trial.

### 10.1.13 Conflict of Interest Policy

The independence of this trial from any actual or perceived influence, such as by the pharmaceutical industry, is critical. Therefore, we will disclose and manage any actual conflict of interest of persons who have a role in the design, conduct, analysis, publication, or any aspect of this trial. Furthermore, persons who have a perceived conflict of interest will be required to have such conflicts managed in a way that is appropriate to their participation in the design and conduct of this trial. The trial leadership, in conjunction with the NIH ECHO office, has established policies and procedures for all trial group members to disclose all conflicts of interest and will establish a mechanism for the management of all reported dualities of interest.

## 10.2 Additional Considerations

N/A

## 10.3 Abbreviations

| AE | Adverse Event |
| --- | --- |
| ANCOVA | Analysis of Covariance |
| CFR | Code of Federal Regulations |
| CLIA | Clinical Laboratory Improvement Amendments |
| CMP | Clinical Monitoring Plan |
| COC | Certificate of Confidentiality |
| CONSORT | Consolidated Standards of Reporting Trials |
| CRF | Case Report Form |
| DCC | Data Coordinating Center |
| DHHS | Department of Health and Human Services |
| DSMB | Data Safety Monitoring Board |
| DRE | Disease-Related Event |
| EC | Ethics Committee |
| eCRF | Electronic Case Report Forms |
| FDA | Food and Drug Administration |
| FDAAA | Food and Drug Administration Amendments Act of 2007 |
| FFR | Federal Financial Report |
| GCP | Good Clinical Practice |
| GLP | Good Laboratory Practices |
| GMP | Good Manufacturing Practices |
| GWAS | Genome-Wide Association Studies |
| HIPAA | Health Insurance Portability and Accountability Act |
| IB | Investigator’s Brochure |
| ICH | International Conference on Harmonisation |
| ICMJE | International Committee of Medical Journal Editors |
| IDE | Investigational Device Exemption |
| IND | Investigational New Drug Application |
| IRB | Institutional Review Board |
| ISM | Independent Safety Monitor |
| ISO | International Organization for Standardization |
| ITT | Intention-To-Treat |
| LSMEANS | Least-squares Means |
| MedDRA | Medical Dictionary for Regulatory Activities |
| MOP | Manual of Procedures |
| MSDS | Material Safety Data Sheet |
| NCT | National Clinical Trial |
| NIH | National Institutes of Health |
| NIH IC | NIH Institute or Center |
| OHRP | Office for Human Research Protections |
| PI | Principal Investigator |
| QA | Quality Assurance |
| QC | Quality Control |
| SAE | Serious Adverse Event |
| SAP | Statistical Analysis Plan |
| SMC | Safety Monitoring Committee |
| SOA | Schedule of Activities |
| SOC | System Organ Class |
| SOP | Standard Operating Procedure |
| UP | Unanticipated Problem |
| US | United States |

# 11 References

1. Feil EG, Baggett K, Davis B, et al. Randomized control trial of an internet-based parenting intervention for mothers of infants. Early Child Res Q 2020;50(Pt 1):36-44. DOI: 10.1016/j.ecresq.2018.11.003.

2. Torres CA, Medina-Kirchner C, O'Malley KY, Hart CL. Totality of the Evidence Suggests Prenatal Cannabis Exposure Does Not Lead to Cognitive Impairments: A Systematic and Critical Review. Front Psychol 2020;11:816. DOI: 10.3389/fpsyg.2020.00816.

3. Gunn JK, Rosales CB, Center KE, et al. Prenatal exposure to cannabis and maternal and child health outcomes: a systematic review and meta-analysis. BMJ Open 2016;6(4):e009986. DOI: 10.1136/bmjopen-2015-009986.

4. Mark K, Desai A, Terplan M. Marijuana use and pregnancy: prevalence, associated characteristics, and birth outcomes. Arch Womens Ment Health 2016;19(1):105-11. DOI: 10.1007/s00737-015-0529-9.

5. Fried PA, Watkinson B, Gray R. Differential effects on cognitive functioning in 13- to 16-year-olds prenatally exposed to cigarettes and marihuana. Neurotoxicol Teratol 2003;25(4):427-36. DOI: 10.1016/s0892-0362(03)00029-1.

6. Fried PA, Watkinson B, Gray R. Differential effects on cognitive functioning in 9- to 12-year olds prenatally exposed to cigarettes and marihuana. Neurotoxicol Teratol 1998;20(3):293-306. DOI: 10.1016/s0892-0362(97)00091-3.

7. Leech SL, Richardson GA, Goldschmidt L, Day NL. Prenatal substance exposure: effects on attention and impulsivity of 6-year-olds. Neurotoxicol Teratol 1999;21(2):109-18. DOI: 10.1016/s0892-0362(98)00042-7.

8. Goldschmidt L, Richardson GA, Larkby C, Day NL. Early marijuana initiation: The link between prenatal marijuana exposure, early childhood behavior, and negative adult roles. Neurotoxicol Teratol 2016;58:40-45. DOI: 10.1016/j.ntt.2016.05.011.

9. Goldschmidt L, Richardson GA, Willford J, Day NL. Prenatal marijuana exposure and intelligence test performance at age 6. J Am Acad Child Adolesc Psychiatry 2008;47(3):254-263. DOI: 10.1097/CHI.0b013e318160b3f0.

10. Campolongo P, Trezza V, Ratano P, Palmery M, Cuomo V. Developmental consequences of perinatal cannabis exposure: behavioral and neuroendocrine effects in adult rodents. Psychopharmacology (Berl) 2011;214(1):5-15. DOI: 10.1007/s00213-010-1892-x.

11. Baggett K, Davis B, Feil E, et al. A Randomized Controlled Trial Examination of a Remote Parenting Intervention: Engagement and Effects on Parenting Behavior and Child Abuse Potential. Child Maltreat 2017;22(4):315-323. DOI: 10.1177/1077559517712000.

12. Ko JY, Coy KC, Haight SC, et al. Characteristics of Marijuana Use During Pregnancy - Eight States, Pregnancy Risk Assessment Monitoring System, 2017. MMWR Morb Mortal Wkly Rep 2020;69(32):1058-1063. DOI: 10.15585/mmwr.mm6932a2.

13. Administration SAaMHS. Key substance use and mental health indicators in the United States: Results from the 2019 National Survey on Drug Use and Health. 2020(HHS Publication No. PEP20-07-01-001, NSDUH Series H-55) (<https://www.samhsa.gov/data/>).

14. Chang JC, Tarr JA, Holland CL, et al. Beliefs and attitudes regarding prenatal marijuana use: Perspectives of pregnant women who report use. Drug Alcohol Depend 2019;196:14-20. DOI: 10.1016/j.drugalcdep.2018.11.028.

15. ElSohly MA, Mehmedic Z, Foster S, Gon C, Chandra S, Church JC. Changes in Cannabis Potency Over the Last 2 Decades (1995-2014): Analysis of Current Data in the United States. Biol Psychiatry 2016;79(7):613-9. DOI: 10.1016/j.biopsych.2016.01.004.

16. ElSohly MA, Chandra S, Radwan M, Majumdar CG, Church JC. A Comprehensive Review of Cannabis Potency in the United States in the Last Decade. Biol Psychiatry Cogn Neurosci Neuroimaging 2021;6(6):603-606. DOI: 10.1016/j.bpsc.2020.12.016.

17. Grant KS, Petroff R, Isoherranen N, Stella N, Burbacher TM. Cannabis use during pregnancy: Pharmacokinetics and effects on child development. Pharmacol Ther 2018;182:133-151. DOI: 10.1016/j.pharmthera.2017.08.014.

18. Falcon M, Pichini S, Joya J, et al. Maternal hair testing for the assessment of fetal exposure to drug of abuse during early pregnancy: Comparison with testing in placental and fetal remains. Forensic Sci Int 2012;218(1-3):92-6. DOI: 10.1016/j.forsciint.2011.10.022.

19. Scheyer AF, Melis M, Trezza V, Manzoni OJJ. Consequences of Perinatal Cannabis Exposure. Trends Neurosci 2019;42(12):871-884. DOI: 10.1016/j.tins.2019.08.010.

20. Tortoriello G, Morris CV, Alpar A, et al. Miswiring the brain: Δ9-tetrahydrocannabinol disrupts cortical development by inducing an SCG10/stathmin-2 degradation pathway. EMBO J 2014;33(7):668-85. (In eng). DOI: 10.1002/embj.201386035.

21. Galve-Roperh I, Palazuelos J, Aguado T, Guzman M. The endocannabinoid system and the regulation of neural development: potential implications in psychiatric disorders. Eur Arch Psychiatry Clin Neurosci 2009;259(7):371-82. DOI: 10.1007/s00406-009-0028-y.

22. Tao R, Li C, Jaffe AE, et al. Cannabinoid receptor CNR1 expression and DNA methylation in human prefrontal cortex, hippocampus and caudate in brain development and schizophrenia. Transl Psychiatry 2020;10(1):158. DOI: 10.1038/s41398-020-0832-8.

23. Wang X, Dow-Edwards D, Anderson V, Minkoff H, Hurd YL. In utero marijuana exposure associated with abnormal amygdala dopamine D2 gene expression in the human fetus. Biol Psychiatry 2004;56(12):909-15. DOI: 10.1016/j.biopsych.2004.10.015.

24. Wang X, Dow-Edwards D, Anderson V, Minkoff H, Hurd YL. Discrete opioid gene expression impairment in the human fetal brain associated with maternal marijuana use. Pharmacogenomics J 2006;6(4):255-64. DOI: 10.1038/sj.tpj.6500375.

25. Thomason ME, Palopoli AC, Jariwala NN, et al. Miswiring the brain: Human prenatal Delta9-tetrahydrocannabinol use associated with altered fetal hippocampal brain network connectivity. Dev Cogn Neurosci 2021;51:101000. DOI: 10.1016/j.dcn.2021.101000.

26. Fried PA, Makin JE. Neonatal behavioural correlates of prenatal exposure to marihuana, cigarettes and alcohol in a low risk population. Neurotoxicol Teratol 1987;9(1):1-7. DOI: 10.1016/0892-0362(87)90062-6.

27. Day N, Cornelius M, Goldschmidt L, Richardson G, Robles N, Taylor P. The effects of prenatal tobacco and marijuana use on offspring growth from birth through 3 years of age. Neurotoxicol Teratol 1992;14(6):407-14. DOI: 10.1016/0892-0362(92)90051-b.

28. Kooijman MN, Kruithof CJ, van Duijn CM, et al. The Generation R Study: design and cohort update 2017. Eur J Epidemiol 2016;31(12):1243-1264. DOI: 10.1007/s10654-016-0224-9.

29. Marchand G, Masoud AT, Govindan M, et al. Birth Outcomes of Neonates Exposed to Marijuana in Utero: A Systematic Review and Meta-analysis. JAMA Netw Open 2022;5(1):e2145653. DOI: 10.1001/jamanetworkopen.2021.45653.

30. Metz TD, Borgelt LM. Marijuana Use in Pregnancy and While Breastfeeding. Obstet Gynecol 2018;132(5):1198-1210. DOI: 10.1097/AOG.0000000000002878.

31. Sacchi C, Marino C, Nosarti C, Vieno A, Visentin S, Simonelli A. Association of Intrauterine Growth Restriction and Small for Gestational Age Status With Childhood Cognitive Outcomes: A Systematic Review and Meta-analysis. JAMA Pediatr 2020;174(8):772-781. DOI: 10.1001/jamapediatrics.2020.1097.

32. Fried PA, Watkinson B, Dillon RF, Dulberg CS. Neonatal neurological status in a low-risk population after prenatal exposure to cigarettes, marijuana, and alcohol. J Dev Behav Pediatr 1987;8(6):318-26. (<https://www.ncbi.nlm.nih.gov/pubmed/3429670>).

33. Richardson GA, Day NL, Goldschmidt L. Prenatal alcohol, marijuana, and tobacco use: infant mental and motor development. Neurotoxicol Teratol 1995;17(4):479-87. DOI: 10.1016/0892-0362(95)00006-d.

34. Jaddoe VW, van Duijn CM, Franco OH, et al. The Generation R Study: design and cohort update 2012. Eur J Epidemiol 2012;27(9):739-56. DOI: 10.1007/s10654-012-9735-1.

35. Fried PA, Watkinson B. 36- and 48-month neurobehavioral follow-up of children prenatally exposed to marijuana, cigarettes, and alcohol. J Dev Behav Pediatr 1990;11(2):49-58. (<https://www.ncbi.nlm.nih.gov/pubmed/2324288>).

36. Navarrete F, Garcia-Gutierrez MS, Gasparyan A, Austrich-Olivares A, Femenia T, Manzanares J. Cannabis Use in Pregnant and Breastfeeding Women: Behavioral and Neurobiological Consequences. Front Psychiatry 2020;11:586447. DOI: 10.3389/fpsyt.2020.586447.

37. Gluckman PD, Hanson MA, Buklijas T. A conceptual framework for the developmental origins of health and disease. J Dev Orig Health Dis 2010;1(1):6-18. DOI: 10.1017/S2040174409990171.

38. Godleski SA, Shisler S, Eiden RD, Huestis MA. Co-use of tobacco and marijuana during pregnancy: Pathways to externalizing behavior problems in early childhood. Neurotoxicol Teratol 2018;69:39-48. DOI: 10.1016/j.ntt.2018.07.003.

39. Ainsworth MD. Infant--mother attachment. Am Psychol 1979;34(10):932-7. DOI: 10.1037//0003-066x.34.10.932.

40. Topping K, Dekhinet R, Zeedyk S. Parent–infant interaction and children’s language development. Educational Psychology 2013;33(4):391-426.

41. Landry SH, Smith KE, Swank PR. Responsive parenting: establishing early foundations for social, communication, and independent problem-solving skills. Developmental psychology 2006;42(4):627.

42. Tamis-LeMonda CS, Kuchirko Y, Song L. Why is infant language learning facilitated by parental responsiveness? Current Directions in Psychological Science 2014;23(2):121-126.

43. Mermelshtine R, Barnes J. Maternal responsive–didactic caregiving in play interactions with 10‐month‐olds and cognitive development at 18 months. Infant and child development 2016;25(3):296-316.

44. Eiden RD, Schuetze P, Shisler S, Huestis MA. Prenatal exposure to tobacco and cannabis: Effects on autonomic and emotion regulation. Neurotoxicol Teratol 2018;68:47-56. DOI: 10.1016/j.ntt.2018.04.007.

45. Eiden RD, Shisler S, Granger DA, Schuetze P, Colangelo J, Huestis MA. Prenatal Tobacco and Cannabis Exposure: Associations with Cortisol Reactivity in Early School Age Children. Int J Behav Med 2020;27(3):343-356. DOI: 10.1007/s12529-020-09875-8.

46. Richardson KA, Hester AK, McLemore GL. Prenatal cannabis exposure-The “first hit” to the endocannabinoid system. Neurotoxicology and Teratology 2016;58:5-14.

47. Schuetze P, Zhao J, Eiden RD, Shisler S, Huestis MA. Prenatal exposure to tobacco and marijuana and child autonomic regulation and reactivity: An analysis of indirect pathways via maternal psychopathology and parenting. Dev Psychobiol 2019;61(7):1022-1034. DOI: 10.1002/dev.21844.

48. Guttentag C.L. P-JC, Landry S.H., Smith K.E., Swank P.R. Individual variability in parenting profiles and predictors of change: Effects of an intervention with disadvantaged mothers. Journal of Applied Developmental Psychology 2006;27:349-369.

49. Landry SH, Smith KE, Swank PR, Guttentag C. A responsive parenting intervention: the optimal timing across early childhood for impacting maternal behaviors and child outcomes. Dev Psychol 2008;44(5):1335-53. DOI: 10.1037/a0013030.

50. Dieterich SE LS, Smith KE, Swank PR, Hebert HM. Impact of Community Mentors on Maternal Behaviors and Child Outcomes. Journal of Early Intervention 2006;28(2):111-124.

51. Landry SH, Smith KE, Swank PR, Zucker T, Crawford AD, Solari EF. The effects of a responsive parenting intervention on parent-child interactions during shared book reading. Dev Psychol 2012;48(4):969-86. DOI: 10.1037/a0026400.

52. Guttentag CL P-JC, Landry SH, Smith KE, Swank PR. Individual variability in parenting profiles and predictors of change: effects of an intervention with disadvantage mothers. Journal of Applied Developmental Psychology 2006;27:349-369.

53. Hatzis D, Dawe S, Harnett P, Barlow J. Quality of Caregiving in Mothers With Illicit Substance Use: A Systematic Review and Meta-analysis. Subst Abuse 2017;11:1178221817694038. DOI: 10.1177/1178221817694038.

54. Lowe J, Qeadan F, Leeman L, Shrestha S, Stephen JM, Bakhireva LN. The effect of prenatal substance use and maternal contingent responsiveness on infant affect. Early Hum Dev 2017;115:51-59. DOI: 10.1016/j.earlhumdev.2017.09.013.

55. Guttentag CL, Landry SH, Williams JM, et al. "My Baby & Me": effects of an early, comprehensive parenting intervention on at-risk mothers and their children. Dev Psychol 2014;50(5):1482-1496. DOI: 10.1037/a0035682.

56. Goodman SH, Garber J. Evidence-Based Interventions for Depressed Mothers and Their Young Children. Child Dev 2017;88(2):368-377. DOI: 10.1111/cdev.12732.

57. Rasga C, Santos JX, Lopes AL, Marques AR, Vilela J, Asif M, Oliveira G, Bennett D, Walker C, Schmidt RJ, Vicente AM. Adapting the ELEAT (Early Life Exposure Assessment Tool) to Portugal - a pilot study to tackle gene-environment interactions in Autism Spectrum Disorder. bioRxiv 2019. DOI: <https://doi.org/10.1101/520593>.

58. Weber A, Miskle B, Lynch A, Arndt S, Acion L. Substance Use in Pregnancy: Identifying Stigma and Improving Care. Subst Abuse Rehabil 2021;12:105-121. DOI: 10.2147/SAR.S319180.

59. Practice CoO. Opioid use and opioid use disorder in pregnancy: Committee opinion. Obstetrics and Gynecology 2017;130(711):e81-e94.

60. Skelton KR, Donahue E, Benjamin-Neelon SE. Validity of self-report measures of cannabis use compared to biological samples among women of reproductive age: a scoping review. BMC Pregnancy Childbirth 2022;22(1):344. DOI: 10.1186/s12884-022-04677-0.

61. El Marroun H, Tiemeier H, Jaddoe VW, et al. Agreement between maternal cannabis use during pregnancy according to self-report and urinalysis in a population-based cohort: the Generation R Study. Eur Addict Res 2011;17(1):37-43. DOI: 10.1159/000320550.

62. Jones HE, Kaltenbach K, Heil SH, et al. Neonatal abstinence syndrome after methadone or buprenorphine exposure. N Engl J Med 2010;363(24):2320-31. DOI: 10.1056/NEJMoa1005359.

63. Kohlasch KL, Cioffredi LA, Lenninger C, et al. Factors associated with parent views about participation in infant MRI research provide guidance for the design of the Healthy Brain and Child Development (HBCD) study. Dev Cogn Neurosci 2021;50:100986. DOI: 10.1016/j.dcn.2021.100986.
