# Supplementary material for: The protocol for a pilot feasibility trial of Improving Neurodevelopmental ouTcomes After prenatal Cannabinoid in uTero exposure (INTACT) study for a multi-center trial
Source: PLoS One. 2025 May 12;20(5):e0322035. doi: 10.1371/journal.pone.0322035 (PMC12068654; doi:10.1371/journal.pone.0322035)
Supplement: Attachment — Submitted filename: Response to Reviewers.docx [file pone.0322035.s004.docx]

To Whom It May Concern:

We are grateful for the feedback received and the opportunity to improve the manuscript submission. Please see our responses below to each of the comments provided. We look forward to your response after the revisions have been reviewed.

**Editor Comments:**

1. Please ensure that your manuscript meets PLOS ONE’s style requirements, including those for file naming.

*We have revised the formatting of the manuscript per the PLOS Study Protocol Articl Template. This included moving the author contributions immediately after the discussion, followed by acknowledgements, supporting information which includes abbreviations, and references. The author contributions were revised to use the CRediT taxonomy descriptions. The availability of the data and materials was updated. The abstract was shortened to meet the 300-word limit.*

2. Thank you for stating the following financial disclosure. Please state what role the funders took in the study. If the funders had no role, please state: The funders had no role in study design, data collection and analysis, decision to publish, or preparation of the manuscript.

*We apologize for the oversight in not originally providing these details. The financial disclosure statement has been revised and now includes the following: “As this study is being conducted under cooperative agreement funding mechanisms, LYF and PA, employees of the National Institute of Health, participated in study design, preparation of the manuscript and the decision to publish. The content is solely the responsibility of the authors and does not represent the official views of the National Institute of Health.” This information has also been added to the cover letter as recommended.*

3. Please amend either the title on the online submission form or the title in the manuscript so that they are identical.

*This oversight has been resolved, with the titles now identical between the online submission form and in the manuscript.*

4. Your ethics statement should only appear in the Methods section of your manuscript.

*The ethics statement has been moved from the end of the manuscript to the methods section as suggested.*

5. Please include a caption for Figure 1, 2, 3 and 4.

*The captions for the Figures have been added to the text as recommended in the guidelines for submission. We apologize for not originally including this information.*

6. Upon checking, your supplementary figure is the same in your figure. Please remove the duplicate figures.

*The supplementary figures that were previously included are the licenses allowing publication that are from BioRender, where the figures were created. The title of these files has been clarified (example - Fig 1 License) to minimize confusion.*

7. Please review your reference list to ensure that it is complete and correct.

*The reference list has been reviewed to ensure completeness and that it is correct.*

**Reviewer Comments:**

1. Is the methodology feasible and described in sufficient detail to allow the work to be replicated? No

*Additional information has been added to the data management section and the statistical analysis section, which should now provide sufficient detail to allow the work to be replicated. Regarding the statistical analysis, the primary goal of this pilot study is to assess the feasibility of the intervention. A single-arm design allows the study team to focus on this aspect without the complexity of a control group. Also, a single-arm design can be more practical and cost-effective. Pilot studies are not typically designed for formal hypothesis testing. A precise sample size calculation is not necessary. In the study protocol, we proposed the sample size based on practical considerations such as the estimated number of potential participants from the sites and the timeline of the study. For each objective, we set quantitative benchmarks for feasibility measures by defining the targeted rates for recruitment, retention, and adherence.*

2. Have the authors described where all data underlying the findings will be made available when the study is complete? No

*We apologize for this oversight and have revised the availability of data and materials. This now reads “In accordance with the NIH Data Management Sharing Policy, the de-identified dataset for this trial will be uploaded at the time of data publication to the NICHD Data and Specimen Hub (DASH), a controlled-access, public use database.”*

3. The data management section of this pilot is rather brief. It needs an expansion of quality control methodology for data collection in the field as well as for central data processing in the main database.

*Per the recommendation, we have expanded the quality control methodology for data collection in the field as well as for central data processing in the main database. We believe that the added information strengthens this section and now meets reviewer expectations.*
